# Facing the challenge of teaching emotions to individuals with low- and high-functioning autism using a new Serious game: a pilot study

**DOI:** 10.1186/2040-2392-5-37

**Published:** 2014-07-01

**Authors:** Sylvie Serret, Stephanie Hun, Galina Iakimova, Jose Lozada, Margarita Anastassova, Andreia Santos, Stephanie Vesperini, Florence Askenazy

**Affiliations:** 1 Autism Resources Center, Child and Adolescent Psychiatry Department, University Hospital CHU-Lenval, Nice, France; 2 Anthropology and Cognitive and Social Psychology Research Unit (LAPCOS, EA 7278), University of Nice Sophia Antipolis, Nice, France; 3 Sensory And Ambient Interfaces Laboratory, CEA LIST/DIASI, Fontenay-aux-Roses, France; 4 Child and Adolescent Psychiatry Department, University Hospital CHU-Lenval, Nice, France

**Keywords:** Serious game, High-functioning Autism, Low-functioning Autism, Social Skills Training, Emotion Recognition, Computer-based Intervention

## Abstract

**Background:**

It is widely accepted that emotion processing difficulties are involved in Autism Spectrum Conditions (ASC). An increasing number of studies have focused on the development of training programs and have shown promising results. However, most of these programs are appropriate for individuals with high-functioning ASC (HFA) but exclude individuals with low-functioning ASC (LFA). We have developed a computer-based game called JeStiMulE based on logical skills to teach emotions to individuals with ASC, independently of their age, intellectual, verbal and academic level.

The aim of the present study was to verify the usability of JeStiMulE (which is its adaptability, effectiveness and efficiency) on a heterogeneous ASC group. We hypothesized that after JeStiMulE training, a performance improvement would be found in emotion recognition tasks.

**Methods:**

A heterogeneous group of thirty-three children and adolescents with ASC received two one-hour JeStiMulE sessions per week over four weeks. In order to verify the usability of JeStiMulE, game data were collected for each participant. Furthermore, all participants were presented before and after training with five emotion recognition tasks, two including pictures of game avatars (faces and gestures) and three including pictures of real-life characters (faces, gestures and social scenes).

**Results:**

Descriptive data showed suitable adaptability, effectiveness and efficiency of JeStiMulE. Results revealed a significant main effect of Session on avatars (ANOVA: F (1,32) = 98.48, *P* < .001) and on pictures of real-life characters (ANOVA: F (1,32) = 49.09, *P* < .001). A significant Session × Task × Emotion interaction was also found for avatars (ANOVA: F (6,192) = 2.84, *P* = .01). This triple interaction was close to significance for pictures of real-life characters (ANOVA: F (12,384) = 1.73, *P* = .057). *Post-hoc* analyses revealed that 30 out of 35 conditions found a significant increase after training.

**Conclusions:**

JeStiMulE appears to be a promising tool to teach emotion recognition not only to individuals with HFA but also those with LFA. JeStiMulE is thus based on ASC-specific skills, offering a model of logical processing of social information to compensate for difficulties with intuitive social processing.

**Trial registration:**

Comité de Protection des Personnes Sud Méditerranée V (CPP): reference number 11.046 (https://cpp-sud-mediterranee-v.fr/).

## Background

Autism Spectrum Conditions (ASC) are characterized by deficits in communication and social interaction as well as by repetitive stereotyped behaviors [1]. These are complex and heterogeneous disorders affecting the quality of reciprocal social interactions, which is one of the most persistent symptoms in ASC [2]. These difficulties are associated with social cognitive difficulties in ASC [3,4] and, thus, are a major source of handicap for these individuals. Undeniably, emotions, conveyed by facial expressions, gestures, words or situations, are critical signals for understanding others’ feelings and intentions and for regulating social interactions [5]. Individuals with ASC show atypical emotion processing. A number of studies have found deficits in this domain, often related to level of intelligence. However, the results of these studies are sometimes controversial [6,7]. These deficits concern not only emotional facial expressions, but also emotional gestures [8], emotional scenes [9] and the recognition of emotions on the basis of contextual cues [10]. Furthermore, individuals with ASC have reduced spontaneous social motivation [11] and shared emotions [12].

The social world is a highly complex system, constantly undergoing major, non-predictable, multi-domain, and random changes. It has been suggested that if rules exist in such a system, they are too complex to be fully understood by individuals with ASC. The same assumption is valid for the integration of these rules in social training [13]. For this reason, the development of social training in ASC has been limited [14-16]. Yet, emotion processing skills in ASC progress over time [17,18], suggesting that individuals with ASC have a certain learning potential [19], possibly based on the development of compensatory strategies [20-22]. Previous studies suggest that using stimuli with relatively reduced complexity, like cartoons, to teach emotions to children and adolescents with ASC is a beneficial therapeutic option. Indeed, there is evidence that emotion processing of real faces may be affected, while emotion processing of cartoon/avatar faces is relatively spared [23-25].

### ICT and ASC

In this context, interventions based on Information and Communication Technologies (ICT) appear to be of special interest as they present several advantages for individuals with ASC: 1) these individuals usually show a strong interest in electronic media and devices [26,27]; 2) these devices operate according to predictable rules and the information provided is clear, structured and unambiguous [28]; 3) ICT do not involve complex socio-emotional expectations [29,30]; and 4) ICT may include virtual or synthetic environments, allowing individuals to experiment with various social situations, while reducing their social anxiety, as well as the fear of failing or of rejection that these individuals with ASC often experience in real face-to-face interactions [31,32].

### Social cognition, ICT and ASC

In the last decade, different ICT-based programs have been developed to teach social skills to individuals with ASC [33,34]. Most of these training programs have targeted emotion recognition and have used photographs, videos, combined visual and audio stimuli or animated emotional expressions of fully or partially disclosed faces [29,35-38]. Other interventions have also combined emotions with stimuli likely to capture the attention and the interest of children with ASC (for example, trains on the *Transporters* DVDs; [39]). These ICT-based programs reported interesting and encouraging results about the possibility to enhance emotion recognition in individuals with ASC. However, they present a number of limitations.

These programs include, for the most part, unimodal or non-integrated isolated stimuli (for example, visual and/or auditory stimuli), a limited number of scenarios (for example, static images or videos) and a considerable verbal demand (oral/written instructions and response options). Human-machine interaction is thus limited and participants with ASC experience difficulties understanding the task and/or responding accordingly. In addition, the reduced flexibility of scenarios does not allow participants to modulate their interaction with the programs (for example, to choose the game’s avatar with which they want to interact, to choose when to interact with it, and to provide an online adjustment of stimulations as a function of the participants’ responses). Finally, the major limitation is that most of these programs were designed for individuals with high-functioning autism (HFA) and thus do not cover the wide spectrum of ASC, which includes around 40 to 60% of individuals with low-functioning autism (LFA) [38,40].

In order to provide a contribution in this direction, we developed an individual, interactive and multi-sensory computer game called JeStiMulE. JeStiMulE aims at teaching emotions not only to children and adolescents with HFA but also to those with LFA. It is a Serious game combining the fun of playing with learning. It includes several exercises to train emotion recognition on avatars (faces, gestures and social scenes), with similar features and goals to those of common video games for children. It also includes motivating instructional aspects (for example, short sequences with an immediate feedback, visual rewards, an innovative vibrotactile gamepad). Moreover, the player has the possibility to create his/her own avatar and to move in a virtual environment. This type of training environment offers the players the opportunity to experiment with various social situations which are similar to real life, to move freely and to choose when and how to interact with other avatars.

Furthermore, the environment developed in JeStiMulE is multi-sensory. Visual, audio and tactile stimulations are provided to facilitate the player’s immersion in the virtual world and to increase the attractiveness of the game. JeStiMulE contains other adaptations which are appropriate to the heterogeneous profile of individuals with ASC (LFA and HFA). Each emotion corresponded to a code. This allows non-verbal children and adolescents with ASC, as well as non-readers, to interact with the game and to be able to learn different emotions. Finally, given that ASC individuals are more efficient in processing the elements of the physical environment than in processing the social and emotional elements [41], JeStiMulE offers an ‘ASC-friendly environment’ where emotional and social elements are linked together by logical rules. According to Baron-Cohen [13], individuals with ASC are extreme ‘systemizers’ and prefer organized environments based on logical rules. Interestingly, logical skills used on JeStiMulE such as analogical reasoning [42-44] and implicit learning [45-47] appear as cognitive peaks in ASC (independent of overall IQ). In addition, these do not necessarily involve verbal or social skills. The aim of JeStiMulE is to compensate difficulties in intuitively understanding the social world through learning strategies adapted to the autistic cognitive profile. In this sense, using logical skills to teach emotions to individuals with ASC seems a relevant therapeutic option.

The present study aims at verifying the usability (which is its adaptability, effectiveness and efficiency) [48] of JeStiMulE, on a heterogeneous group of individuals with ASC. We hypothesized that after a four-week training with JeStiMulE, a performance improvement would be found in emotion recognition tasks, including not only on game avatars (faces and gestures) but also on pictures of real-life characters (faces, gestures and social scenes).

## Methods

### Participants

Thirty-six children and adolescents were recruited by the Autism Resources Center (University Hospital of Nice, France) in four day-care units. All participants received a diagnosis of ASC based on the *Diagnostic and Statistical Manual of Mental Diseases, Fourth Edition* (DSM-IV-R) [1] criteria for ASC, as well as on the Autism Diagnostic Interview-Revised (ADI-R) [49] and/or the Autism Diagnostic Observation Schedule (ADOS) [50]. Three participants were excluded for the following reasons: 1) non-efficient use of the gamepad (N = 2), and 2) refusal to play (N = 1). The inclusion group included 33 participants. The participants’ characteristics are presented in Table 1 (see also Additional file 1 for individual details). IQ was assessed using the Wechsler Abbreviated Scale of Intelligence (WASI) [51]. Semantic-syntactic level (age) was assessed using the ECOSSE (*Epreuve de Compréhension Syntaxico*-*SEmantique*) [52] and reading ability was assessed using the ALOUETTE [53]. Information regarding schooling and special care (educative and/or therapeutic) was also collected for each participant.

**Table 1:** **Participants**’ **characteristics**

**Characteristic (N** = **33)**	**Number**	
Gender	31 male, 2 female	
Autism	23	-
Asperger syndrome	4	-
PDD-NOS	6	-
Readers	19	-
Non-readers	14	-
	Mean score (Standard deviation)	Range
Age (years)	11.4 (3.16)	6 to17
WASI	70.5 (27.6)	35 to 129
Age of semantic-syntactic language (years)	4 (4.7)	< 1 to 12
Schooling (hours/week)	15.1 (9.7)	0 to 28
Special care (hours/week)	10.6 (10.1)	0 to 28

PDD-NOS, Pervasive Developmental Disorder-Not Otherwise Specified; WASI, Wechsler Abbreviated Scale of Intelligence.

Only participants who were able to discriminate primary and secondary colors and had already used a computer were included in the training. Informed consent was obtained from all participants and their parents prior to participation. All procedures were approved for all day-care unit partners by the Local Ethical Committee (Comité de Protection des Personnes Sud Méditerranée V: reference number 11.046).

## Materials

### JeStiMulE: game description

**Figure 1:**
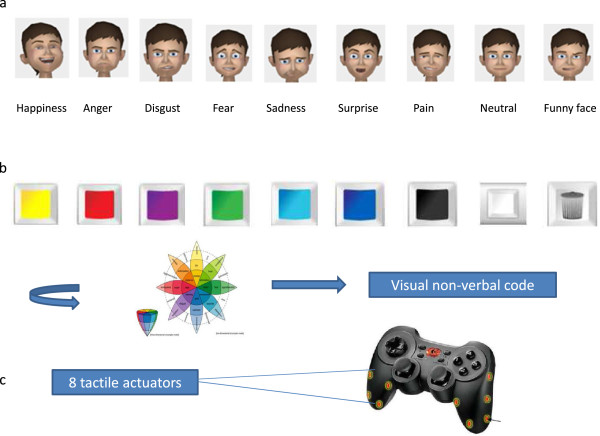
**Illustration of JeStiMulE’s content and equipment. (a)** Facial expressions, **(b)** Visual non**-**verbal code, **(c)** Gamepad with actuators.

JeStiMulE is the prototype of an individual interactive and multi-sensory computer game played with a gamepad (see Additional file 2 for a video trailer of JeStiMulE). It was specifically designed for children and adolescents with ASC (HFA and LFA). It aims at training emotion recognition skills, including facial expressions, emotional gestures, and social situations. For this purpose, nine expressions are presented in the game: six basic emotions (which are happiness, anger, disgust, fear, sadness, surprise), one complex emotion (that is pain) and two complementary expressions (which are neutral and ‘funny face’). These emotions are displayed on both static and animated avatars. The expressions of pain were included in order to promote the development of empathy [54]. Furthermore, complementary expressions were included to facilitate the distinction between emotional and non-emotional expressions. This is particularly important for children and adolescents with ASC without functional language, to whom a verbal explanation of this distinction is often inefficient. In this way, a face without emotional expression corresponded to a neutral/non-emotional facial expression and a ‘funny face’ reflected an intentional inappropriate facial expression. Each expression was associated to one facial expression and three gestures. Each facial expression was different from another by mouth, eyes and eyebrows shape, opening or tilt. Only one emotional valence was presented for each emotion (see Figure 1a).

JeStiMulE is a virtual reality game with a multi-sensory environment. Visual, tactile, and auditory stimulations are provided to facilitate game immersion. In JeStiMulE, each expression was associated to a visual non-verbal code (see Figure 1b), a corresponding verbal written code (which comprises emotional words and idiomatic expressions), and a tactile pattern (see further details below). Visual non-verbal codes corresponded to colors and a symbol. Each basic emotion was associated to a specific color from Plutchick’s emotional wheel (happiness = yellow, anger = red, disgust = purple, fear = green, sadness = light blue, surprise = dark blue) [55] (see Figure 1b). Pain was associated with black. Neutral was associated with the color white and ‘funny face’ was associated with a trash can. This last choice was made to introduce the notion of inappropriate facial expressions, in particular to participants without functional language.

Emotional words corresponded to the literal description of each emotion. Idiomatic expressions corresponded to short sentences that have a figurative meaning conventionally understood by native speakers. This meaning is different from the literal meaning of the idiomatic expression’s individual elements. Idiomatic expressions are very common in everyday language and constitute one essential part of the human’s emotional communication [56]. For example, the emotion of Fear may be expressed figuratively by the idiomatic expression ‘to get cold feet’ (French: ‘*avoir la chair de poule’* = ‘having the chicken skin’ (literal meaning)). These expressions were used because their meaning requires individuals with ASC to learn them explicitly [57].

Each emotion was associated to a specific tactile pattern to promote the association between emotions and a physical imprint. Each tactile pattern could be considered as a word in an emotional language corresponding to an icon in visual communication [58,59]. Tactile patterns reinforce emotional meaning. The patterns were developed in a number of iterative user tests. The tactile stimulations were produced by eight actuators distributed over the gamepad body (see Figure 1c). The actuators position was defined by measuring the contact zones between the hand and the gamepad.

Auditory stimulations were repetitive musical sequences, emotional onomatopoeias and environmental noises presented along and according to the social scenes. JeStiMulE was designed based on specific user requirements. In order to offer adapted response options to all participants, in JeStiMulE each expression was presented not only with its corresponding non-verbal code but also with two corresponding verbal written codes (emotional words and idiomatic expressions).

**Figure 2:**
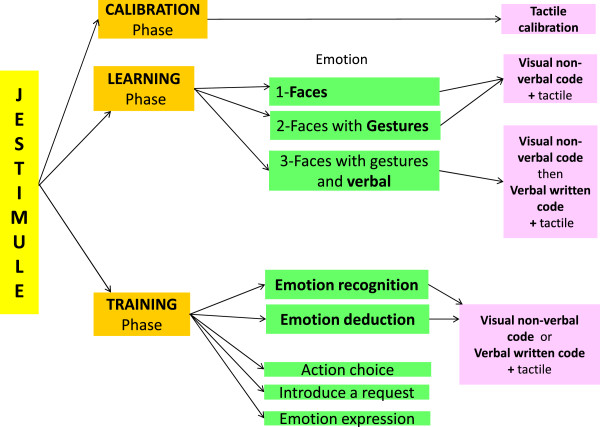
**Illustration of JeStiMulE’s design.**

JeStiMulE did not include verbal instructions (either oral or written). Its design allowed participants to discover the game either intuitively or using a trial and error strategy. In order to favor the development of interpersonal interactions, JeStiMulE was conceptualized as a tool to be used by a player accompanied by a caregiver. The caregiver could thus help the player, either verbally or with gesture and physical guidance.JeStiMulE comprised three phases (which are calibration, learning and training, see Figure 2).

**Figure 3:**
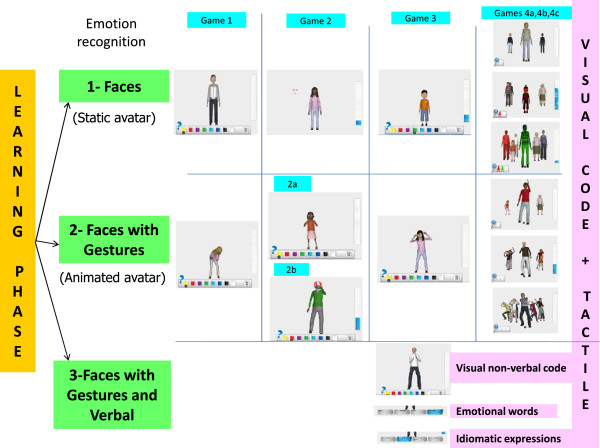
Illustration of the learning phase.

1) *JeStiMulE calibration phase*: a tactile calibration was conducted separately for each participant according to his/her tactile sensory profile measured by the DUNN parental questionnaire [60]. Based on this, participants with a hypersensitivity profile were given a weak tactile stimulation, the ones with normal sensitivity were given a medium tactile stimulation and the ones with a hyposensitivity profile were given a strong tactile stimulation. This phase also included a user-test to ensure that participants were able to use the gamepad to move the avatar around the platform.

2) *JeStiMulE learning phase* (see Figure 3): this phase included three levels with gradually increasing complexity. On level 1, emotions were displayed on faces and associated with a visual non-verbal code and a specific tactile stimulation. On level 2, emotions were displayed on faces combined with gestures and associated with a visual non-verbal code and a specific tactile stimulation. On level 3, emotions were displayed on faces combined with gestures and associated with a visual non-verbal code, a verbal written (emotional words and idiomatic expressions) code and a specific tactile stimulation.

Participants were first trained to recognize different emotional faces and gestures in static avatars and then in animated avatars. Avatars had different identities, ages and clothes. They were presented full-body, first alone and then in groups (of three, six or nine avatars).

In game 1, participants discovered different emotions. First, they only saw the visual non-verbal code on the screen. They used the gamepad’s joystick to move the cursor horizontally in two directions (which were right and left), across the different colors of the visual non-verbal code. When a participant clicked on a color, a full-body avatar, with the chosen expression (first facial, then facial combined with gesture expressions), appeared on the screen. Participants were free to choose the color they wanted to click on. On the gamepad, only the joystick and one button were activated to facilitate their use. Participants switched automatically to the next game after having seen each emotion ten times. Participants learned to make one-to-one associations. These associations started with a reduced level of complexity (for example, emotional face - code). Complexity increased with the gradual introduction of static then animated avatars, of additional emotional cues (which were faces + gestures), as well as by diversifying the avatars’ identities, ages and clothes.

In game 2, the participants’ emotional expression recognition was tested. On the first level, a static avatar with a facial expression appeared on the screen. The participants had to click on a moving target featuring the avatar’s eyes and mouth. This was done in order to guide the participants’ gaze towards the avatar’s face. Then, the visual non-verbal code appeared on the bottom of the screen. The participants could then choose the correct color corresponding to a given emotion. They switched to the next game after having succeeded three out of ten emotional recognitions for each emotion presented. On the level 2, an animated avatar appeared on the screen with a facial expression combined with an inappropriate gesture (game 2a) and a masked face with an appropriate gesture (game 2b). Following this, the visual non-verbal code appeared and the participants had to choose the correct responses. As described above, the participants switched to the next game after having succeeded in three out of ten emotional recognitions for each emotion presented. The participants learned to establish priority rules in order to successfully extract the relevant emotional information (for example, focus on eyes and mouth; focus on face if gestures were inappropriate; and focus on gestures if the face was masked).

In game 3, the participants’ learning and acquisition were tested. They switched to the next game after having succeeded in three out of ten emotional recognitions for each emotion presented. It is important to note that, in contrast with the other levels, level 3 of game 3 was exclusively dedicated to children and adolescents with HFA and AS. The recognition of emotion was performed on all avatars (facial and gestural expressions) of the game. It involved three consecutive responses modalities (visual non-verbal code, verbal written code with emotional words and idiomatic expressions).

Finally, game 4 (called ‘odd one out’ game) introduced multiple avatars with different emotions in order to promote emotion categorization. Participants scrolled down avatars (on an invisible carousel) with the gamepad joystick. They clicked on one, two or three avatars which did not express the same emotion as the others. In this game, there were three levels: one odd one out of three avatars (game 4a), two odd ones out of six avatars (game 4b) and three odd ones out of nine avatars (game 4c).

During each game of the learning phase, the participant could monitor his/her performance on a vertical colored gauge showing their progression. Feedback is provided after each trial in all games. Positive and negative feedback were provided by a brief and subtle green or red flash. To summarize, this learning phase offered a structured, progressive and adapted learning procedure, involving implicit learning, visual discrimination, attention to detail, categorization and memory skills.

**Figure 4:**
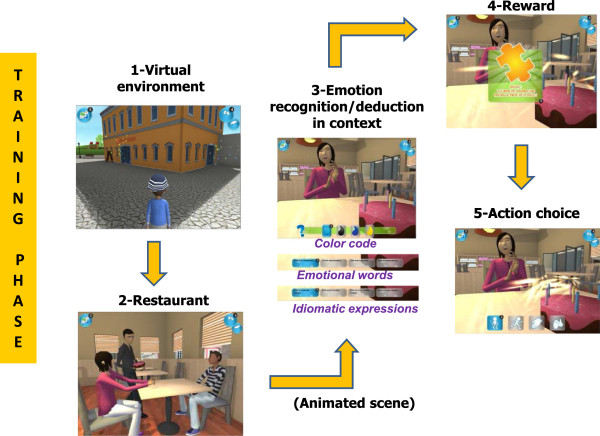
**Illustration of the training phase (part 1).**

**Figure 5:**
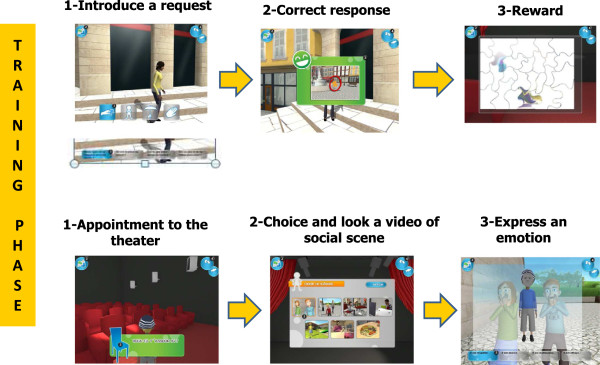
Illustration of the training phase (part 2).

3) *JeStiMulE training phase* (see Figures 4 and 5): the game was developed using a 3D real time mechanism - based on Unity 3D (Unity Technologies https://unity3d.com/company).

The training phase included three modules in which the participants could apply the knowledge acquired during the learning phase of the game. Each module was composed of the same scenarios (which were 26 social scenarios and 4 scenarios involving a request formulation). The modules also comprised a puzzle (see further details below).

**Figure 6:**
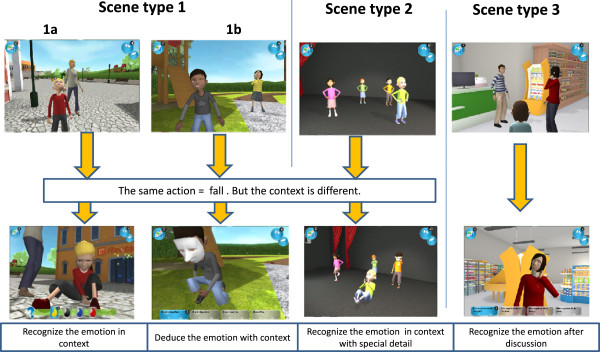
**Example of scenes used in JeStiMulE. (1a)** Recognize the emotion in context, **(1b)** Deduce the emotion with context, **(2)** Recognize the emotion in context with special detail, **(3)** Recognize the emotion after discussion.

Before starting, the participant had to choose and personalize his/her own avatar. The personalization of the avatar included its gender, hair and eye color, its clothes, and its accessories. The training took place in a virtual environment (a city), where the participant’s avatar could move freely in five different areas, which were a square, a garden, a restaurant, a theatre and a shop. The participant could choose where and when his/her avatar would interact with the other avatars in the game. He/she could also choose to play with objects present in the environment (for example, a ball, a menu). When the participant’s avatar approached another avatar, an active social scene was automatically initiated. All the scenes were animated as video game event scenes (that is as cut scenes). Once the social scene was finished, the participant had to identify the emotion expressed in the scene (either by recognition or by deduction). Only one response modality (a color code, emotional words or idiomatic expressions) was available, in accordance with each participant’s profile. For all participants, tactile stimulations were associated with the chosen response modality.For each correct response, the participant gained a piece of the puzzle. The participant’s goal was to complete the puzzle which comprised 30 pieces. After obtaining each puzzle piece, the participant had to choose an action among four visual supports (see Figure 4). Once he/she completed the puzzle, he/she could go to the theatre, sit down, watch his/her favorite scene and imitate the presented emotion (see Figure 5). All scenes with incorrect responses appeared randomly in successive trials.The scenes comprised seven expressions (six basic emotions and pain). Figure 6 provides an example illustrating the logical progression of the scenes. It was a probabilistic progression, ranging from the most probable associations to exceptions.First, an action was associated to the most probable expression (for example, falling - pain). This was the case in two types of scene, which are in one with an unmasked face (see Figure 6.1a) and in another with a masked face (see Figure 6.1b). These two scenes only differed from each other in terms of slight context variations (for example, number and identity of avatars, area). Thus, these scenes presented a high level of similarity, allowing participants to deduce the expression displayed on the masked face, by analogy with the expression displayed on the unmasked face shown before.Then, the action appeared in another scene, where it was associated to a less probable, but still coherent with the context, expression (for example, falling - surprise at the theatre, see Figure 6.2). In this case, the aim was to provide examples of particular situations (exceptions to the rule presented before).Finally, the action was omitted and the participant had to recognize the expression (for example, pain) on the basis of non-verbal cues (for example, physical elements of the context) during a conversation between avatars (see Figure 6.3). Note that in this last case, the content of conversation was not perceptible. For this reason, the participant had to use his/her attention skills to detail and base his/her judgments on non-verbal cues only.

In order to promote the transfer of skills from one context to another (based on analogical reasoning), several variations were introduced from one module to the other within the same scenarios. Furthermore, the fact that modules followed a probabilistic progression encouraged participants to deduce rules, based on implicit learning, from one scene to the other. Thus, JeStiMulE is based on ASC-specific skills and offers a model of logical processing of social information in order to compensate their difficulties with intuitive social processing.

### JeStiMulE: assessment

The usability assessment of JeStiMulE was based on three criteria: the adaptability, the effectiveness and the efficiency [61].

### Adaptability

JeStiMulE design paid special attention to the sensory and learning profile of ASC in order to allow a heterogeneous (age, IQ, reading, and so on) group of individuals with ASC to play. It included the following adaptations:

(1a) a possibility to choose the intensity of tactile stimulations (strong, medium, weak and inactive) in accordance with each participant’s sensory profile;

(1b) a learning phase based on a learning-by-association procedure (visual non-verbal code-expression during the learning phase);

(1c) a possibility to choose a user-adapted response option (color, emotional words or idiomatic expressions) in accordance with each participant’s age, level of semantic-syntactic language acquisition or reading abilities;

(1d) games adapted to the cognitive style of the individuals with ASC: matching, attention to detail, implicit learning, categorization, analogical reasoning. For each game, a performance criterion was pre-established.

### Effectiveness

JeStiMulE includes two key-stages (visual non-verbal code-expression associations and recognition of emotions displayed in social scenes) which participants had to achieve. This structured design aimed at ensuring that the participants understood the principles of the game and could play efficiently.

### Efficiency

The goal of JeStiMulE is to help participants to recognize emotions displayed in social scenes. The participants who achieved the two key-stages have been considered to have completed the main goal of the game, which is playing with effectiveness during a limited time.

### Experimental tasks

**Figure 7:**
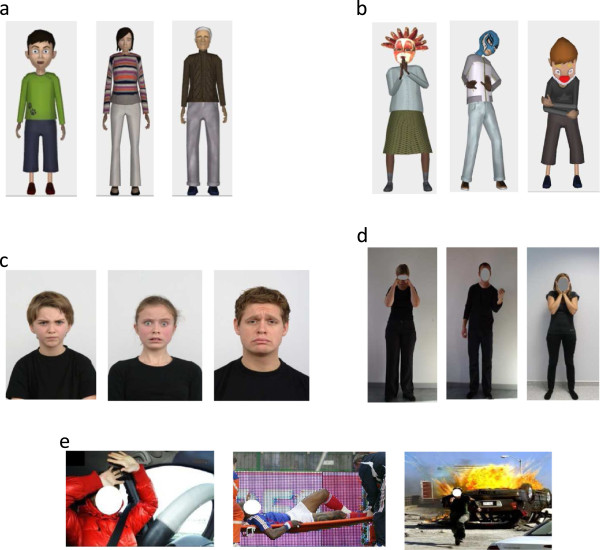
**Example of the stimuli used in the experimental tasks. (a)** Avatar faces. **(b)** Avatar gestures. **(c)** Real-life character faces. **(d)** Real-life character gestures. **(e)** Real-life social scenes.

In order to assess the acquisition of emotional skills and progression after playing JeStiMulE, all participants were presented with five emotion recognition tasks before and after training. The tasks comprised 2D visual stimuli (photographs) and were separated into two types: 1) emotions displayed by JeStiMulE’s avatars (faces and gestures), and 2) emotions displayed by real-life characters (faces, gestures and social scenes). Seven emotions were presented in each task. Stimuli examples for all tasks are presented in Figure 7.

The total number of trials was 154, corresponding to:Twenty-one avatar faces (three trials for each emotion) presented in a full-body display (see Figure 7a).Twenty-one avatar gestures (three trials for each emotion) presented in a full-body display on which faces were masked to make participants focus on the emotional gestures (see Figure 7b).

Thirty-nine real-life character faces (six trials for each basic emotion + three for pain^a^, see Figure 7c) taken from the Radbout Faces Database [62].Forty-two real-life character gestures (six trials for each emotion) presented in a full-body display where faces were masked by a grey circle to make participants focus on the gestures (see Figure 7d).

Twenty-eight real-life character social scenes (four trials for each emotion; see Figure 7e). Photographs were selected by two independent experts. The characters’ faces were masked in order to make the participants focus on contextual cues to recognize the emotion expressed in the given social situation [10].

In order to verify these tasks reliability, a preliminary data collection was conducted on typically developing individuals (n = 17). Mean results are presented in Additional file 3.

At the beginning of each trial, the stimuli were displayed in the left side of the screen and the response options were displayed in the right side. The response options corresponded to seven rectangles of different colors according to the different expression-color associations included in JeStiMulE. In addition, the word corresponding to the expression was displayed inside each rectangle and every expression was read aloud. Participants were asked to determine the expression displayed by choosing and clicking (mouse click) on the rectangle corresponding to it. Participants were tested individually in a quiet room with pauses, if necessary. They were seated in front of a computer screen. The stimuli were presented in E-Prime 2 (Psychology Software Tools, Inc. http://www.pstnet.com/eprime.cfm), in successive blocks. Each task was presented separately. The order of the trials and the tasks was randomly assigned across participants. Correct responses were scored ‘1’ and incorrect responses or no response were scored ‘0’.

### Procedure

**Figure 8:**
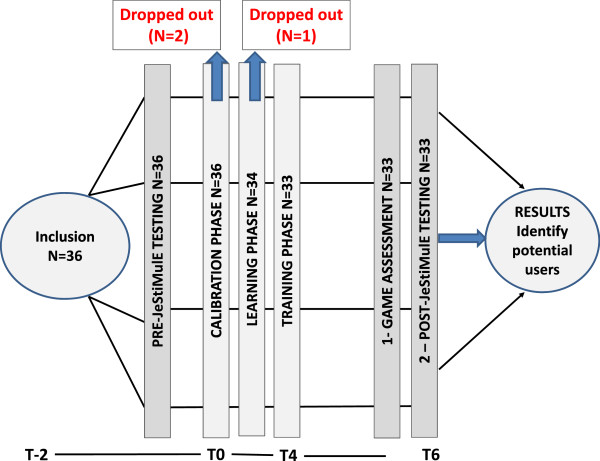
Procedure.

The study procedure is illustrated in Figure 8. After inclusion, all participants were tested on emotion recognition (pre-JeStiMulE testing, two weeks before training). Following this, they were presented with JeStiMulE, starting with a calibration phase, followed by a learning and a training phase.

All sessions were conducted with a caregiver and took place in the same room. Each training session lasted one hour maximum. Participants played twice a week until they completed JeStiMulE. They had four weeks (corresponding to eight sessions) maximum to achieve this goal. The number of sessions thus varied from one participant to the other, according to their performance. Each game level could only be performed once.

In order to verify JeStiMulE’s usability (adaptability, effectiveness and efficiency), game data were collected for each participant.

After JeStiMulE, all participants were re-tested on emotion recognition (post-JeStiMulE testing, two weeks after training).

### Statistical analyses

Descriptive analyses were conducted to verify JeStiMulE adaptability, effectiveness and efficiency.

Data on JeStiMulE’s avatars were analyzed using a repeated measures analysis of variance (ANOVA) (using Statistica 7 device, Statsoft http://www.statsoft.com) including Session (Before versus After), Task (Facial versus Gestures emotion) and Emotion (Happiness versus Surprise versus Sadness versus Fear versus Disgust versus Anger versus Pain). Analyses were completed by a *post-hoc* Tukey HSD test.

Data on real-life characters were analyzed using a repeated measures ANOVA including Session (Before versus After), Task (Facial versus Gestures versus Situations) and Emotion (Happiness versus Surprise versus Sadness versus Fear versus Disgust versus Anger versus Pain). Analyses were also completed by a *post-hoc* Tukey HSD test.

## Results and discussion

### JeStiMulE: assessment

The results of the descriptive analyses conducted to assess JeStiMulE adaptability, effectiveness and efficiency are presented in Tables 2, 3 and 4, respectively (see also Additional file 4).

**Table 2:** **JeStiMulE**’**s adaptability**

**Adaptability**		**% of individuals in the sample**
(1a) Play with tactile stimulation		100
	Strong	15
	Medium	48
	Weak	36
	Inactive	0
(1a) Move the avatar around the platform		100
(1b) Learning by association (learning phase)		-
	Visual non*-*verbal code - faces	91
	Visual non*-*verbal code - gestures	79
(1c) User**-**adapted response options (training phase)		-
	Visual non*-*verbal code (that is color code)	43
	Emotional words	39
	Idiomatic expressions	18
(1d) Games adapted to the ASC cognitive style		-
	Matching games (Games 1.2, 1.3 and 2.3)	83
	Attention to detail (Game 1.2)	91
	Implicit learning (Games 1.2*,* 2.2a and*,* 2.2b)	80
	Categorization (Games 1.4a*,* b*,* c*;* and 2.4a*,* b*,* c)	80
	Analogical reasoning (Modules 1*,* 2 or 3)	91

**Table 3:** **JeStiMulE**’**s effectiveness**

**Effectiveness**				**% of individuals in the sample**
Learning visual non**-**verbal code**-**emotions associations				-
Validation	Visual non*-*verbal code - faces			91
Validation	Visual non*-*verbal code - gestures			79
Recognition of emotions displayed in social scenes (with user**-**adapted response options)				-
	Visual non-verbal code (%)	Emotional words (%)	Idiomatic expressions (%)	-
Playing (training phase)	43	39	18	100
Validation module 1	33	39	18	91
Validation module 1 *+* 2	27	39	18	85

**Table 4:** **JeStiMulE**’**s efficiency**

**Two key**-**stage validation (73% of participants)**	**Mean score (standard deviation)**	**Range**
Number of sessions	6 (1.68)	3 to 8
Session time (minutes)	49 (8.32)	30 to 60

Adaptability (Table 2) - Results indicate that:

1a) all participants were able to use the tactile gamepad and to move the avatar within the virtual world;

1b) as predefined, all participants were trained to the visual non-verbal code-expression associations during the learning phase. Ninety-one percent of the participants were able to learn by association;

1c) during the training phase, 43% of the participants responded using the visual non-verbal code, while 39% of them used emotional words and 18% used idiomatic expressions. All participants found a response modality adapted to their profile;

1d) all ASC-adapted games could be played by at least 80% of the total number of participants.

Effectiveness (Table 3) - After the learning phase, 91% of the participants were able to make JeStiMulE visual non-verbal code-expression *faces* associations and 79% of them were able to make JeStiMulE visual non-verbal code-expression *gestures* associations. After the training phase, 91% of the participants completed JeStiMulE module 1, 85% completed module 1 and 2, and 73% completed all modules, independently of their response modality.

Efficiency (Table 4) - These data are based only on the performance of participants who completed the two key stages of JeStiMulE (which are visual non-verbal code-expression associations and recognition of emotions displayed in social scenes). These participants (73%) played, on average, 6 sessions (range 3 to 8 sessions) of 49 minutes (range 30 to 60 minutes) each.

### JeStiMulE: training effect

#### Pictures of JeStiMulE’s avatars

Individual data are illustrated in Additional file 5. Results of statistical analyses revealed a significant main effect of Session (F (1,32) = 98.48, *P* < .001), Task (Faces versus Gestures, F (1,32) = 5.65, *P* = .02) and Emotion (F (6,192) = 18.60, *P* < .001). These results indicate that participants were more accurate at recognizing emotions after JeStiMulE (M = 64.65, SD = 17.50 versus M = 31.16, SD = 13.34; *P* < .001) and that their overall performance was superior on the Facial (M = 50.72, SD = 15.93) than on Gestural (M = 45.09, SD = 13.79; *P* = .02) emotion recognition task. Moreover, results revealed that emotions such as Sadness (M = 61.87, SD = 10.59), Happiness (M = 60.35, SD = 8.88) and Anger (M = 54.29, SD = 9.79) were better recognized than emotions such as Disgust (M = 33.84, SD = 8.30; all *P* < .001), Pain (M = 38.64, SD = 7.80; all *P* < .001) and Surprise (M = 40.15, SD = 7.72; all *P* < .005).

**Figure 9:**
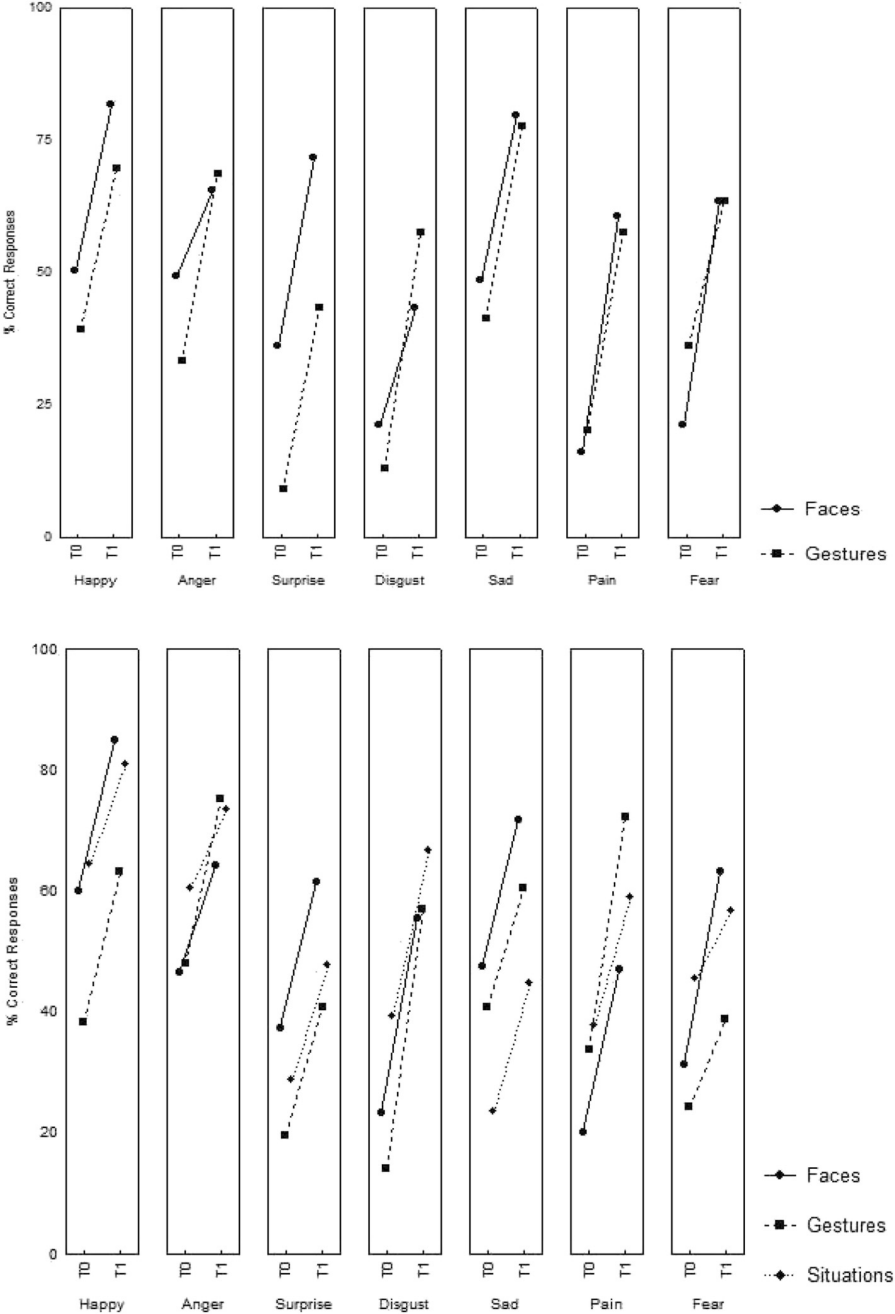
**JeStiMulE’s results.** Percentage of correct responses found on avatars (top) and on photographs of real-life characters (bottom) before (T0) and after (T1) training for each Emotion (happy, anger, surprise, disgust, sad, pain and fear) in each Task (avatar faces and gestures (top); real-life character faces, gestures and social situations (bottom)).

Finally, results revealed a significant Session × Task × Emotion (F (6,192) = 2.84, *P* = .01) interaction. *Post-hoc* analyses revealed that all conditions, except Anger recognition on the faces task, were significant (see Table 5 and Figure 9).

**Table 5:** **Results obtained for JeStiMulE**’**s avatars and for real**-**life characters**

**ASC (N** = **33)**		**Avatar tasks**			**Real**-**life character tasks**		
		**Pre-training**	**Post-training**	**-**	**Pre-training**	**Post-training**	**-**
		**Mean (standard deviation)**		***P***	**Mean (standard deviation)**		***P***
Face	Happy	50.5 (6.5)	81.8 (5.4)	<.001	60.1 (7.4)	84.8 (6.1)	<.001
	Anger	49.5 (7)	65.7 (6.1)	.47 NS	46.5 (6.2)	64.1 (6.4)	.03
	Surprise	36.4 (7.2)	71.7 (6.2)	<.001	37.4 (7.1)	61.6 (6.4)	<.001
	Disgust	21.2 (6.1)	43.4 (6.7)	.02	23.2 (5.8)	55.6 (7.3)	<.001
	Sadness	48.5 (7)	79.8 (6.5)	<.001	47.5 (6.7)	71.7 (6.2)	<.001
	Pain	16.2 (4.4)	60.6 (6.4)	<.001	20.2 (5.2)	47 (6.5)	<.001
	Fear	21.2 (5)	63.6 (6.5)	<.001	31.3 (6)	63.1 (6.7)	<.001
Gesture	Happy	39.4 (6.5)	69.7 (6.2)	<.001	38.4 (5.5)	63.1 (5.3)	<.001
	Anger	33.3 (5.6)	68.7 (6.1)	<.001	48 (7)	75.3 (6.1)	<.001
	Surprise	9.1 (3.6)	43.4 (6.1)	<.001	19.7 (5)	40.9 (5.5)	<.001
	Disgust	13.1 (3.2)	57.6 (6.8)	<.001	14.1 (4)	57.1 (6.2)	<.001
	Sadness	41.4 (5.8)	77.8 (6.1)	<.001	41 (6.3)	60.6 (5)	.004
	Pain	20.2 (4.3)	57.6 (6.4)	<.001	33.8 (5.3)	72.2 (5.7)	<.001
	Fear	36.4 (6)	63.6 (6.5)	<.001	24.2 (5)	38.9 (4.3)	.26 NS
Social scene	Happy	-	-	-	64.4 (6.8)	81.1 (6.3)	.069 NS
	Anger	-	-	-	60.6 (7)	73.5 (6.2)	.058 NS
	Surprise	-	-	-	28.8 (5.6)	47.7 (5.7)	.009
	Disgust	-	-	-	39.4 (6.9)	66.7 (7)	<.001
	Sadness	-	-	-	23.5 (4.5)	44.7 (6.1)	<.001
	Pain	-	-	-	37.9 (6.4)	59.1 (6)	<.001
	Fear	-	-	-	45.5 (6.3)	56.8 (5.4)	.85 NS

#### Pictures of real-life characters

Individual data are illustrated in Additional file 5. Results of statistical analyses revealed a significant main effect of Session (F (1,32) = 49.09, *P* < .001), Task (Faces versus Gestures versus Situations, F (2,64) = 7.93, *P* < .001) and Emotion (F (6,192) = 23.88, *P* < .001). In line with the results for pictures of JeStiMulE’s avatars, these results show that participants were more accurate at recognizing emotions after using JeStiMulE (M = 61.21, SD = 28.83 vesrus M = 37.39, SD = 20.96, *P* < .001). Moreover, results indicate that the overall performance was inferior on the Gestures (M = 44.80, SD = 14.76) than on the Faces (M = 51.01, SD = 17.31, *P* = .007) and the Situations (M = 52.11, SD = 18.22, *P* = .001) tasks. Regarding the Emotion effect, the results of the *post-hoc* analysis revealed that the overall performance on Happy (M = 65.32, SD = 13.35) and Angry (M = 61.32, SD = 13.56) trials was superior compared to the performance on trials including other emotions (M_Surprise_ = 39.35, SD = 11.38; M_Disgust_ = 42.68, SD = 10.87; M_Pain_ = 48.14, SD = 10.80; M_Fear_ = 43.31, SD = 11.11; all *P* < .001).

Finally, the triple Session × Task × Emotion interaction was only close to significance (F (12,384) = 1.73, *P* = .057). In order to further track this interaction, *post-hoc* analyses were conducted. Results are fully presented in Table 5 (see also Figure 9 and Additional file 5).

The present study had two major objectives. Firstly, it aimed at verifying the usability of JeStiMulE (which is its adaptability, effectiveness and efficiency) on a heterogeneous group of individuals with ASC. Secondly, it aimed at investigating whether a four-week JeStiMulE training would improve emotion recognition not only on game avatars (faces and gestures) but also on pictures of real-life characters (faces, gestures and social scenes).

Results indicate that JeStiMulE presents a suitable usability. Most participants were able to play (effectiveness) and to complete the training within the expected time (efficiency), which supports the idea that JeStiMulE specificities, including sensory, cognitive and motivational dimensions, were adapted to the ASC profile. Moreover, participants were not only able to play but they also benefited from the training, as indicated by their improved performance on emotional recognition tasks. The results of this study thus provide evidence of the potential of JeStiMulE for individuals with ASC with heterogeneous intellectual, verbal and academic levels.

As every participant was tested before and after training, he/she would serve as his/her own control. This experimental decision helped us evaluate the effectiveness of the training on each individual. In this study, emotion recognition skills were assessed before and after four-week JeStiMulE training. A significant improvement was found in most of the tasks despite the heterogeneity of the group of participants. To date, most of the studies conducted in this field have focused on homogeneous groups with ASC. Very often, these studies focused on individuals with HFA including AS. There are few studies that include individuals with LFA. For example, Hopkins *et al*. [38] assessed the efficacy of ‘FaceSay’, a computer-based social skills training program for children with LFA and HFA. Their results suggested that providing children with ASC with a controlled, structured, and interactive environment with avatar assistants can help them to improve their social skills. Yet, this improvement was most significant for children with HFA. According to the authors, ‘it is possible that the children with LFA did not completely understand the concepts or directions in the games, and therefore, did not fully benefit from the intervention’ [38].

The present study provides an advance in this research field, by showing that children and adolescents with heterogeneous ASC were able to understand, play and complete JeStiMulE as well as benefit from the intervention. A performance increase was found not only for the pictures of avatars conditions, but also for the tasks including photographs of real-life stimuli (faces, gestures and social situations) with which participants were not trained.

While individuals with LFA and HFA do differ in many features (for example, IQ profile), they all share a fundamental one, that is their interest and preference for rule-based systems and their ease when interacting with them. JeStiMulE was developed specifically to provide a rule-based computer game relying on ‘autistic intelligence’ to develop other, more social, skills. The underlying working hypothesis was thus that rule-based learning could be a relevant pathway to reach gradually more complex, social learning in ASC. The idea that rule-based environments could compensate for difficulties in the domain of emotion recognition in ASC [39] is not novel. When designing JeStiMulE, we assumed that individuals with ASC could use their logical skills to learn emotions and could evolve in a systemizing environment.

JeStiMulE uses a virtual environment to simulate different social situations, offering thus more ecological learning opportunities than those of devices using static stimuli [26].

Participants included in this study only played once each game level and none of them repeated a successfully completed level or module. These training criteria clearly differ from that of more traditional approaches using repetitive learning to develop skills. For instance, behavioural methods encourage a ratio of *known*: *new tasks* of around 80:20% [63]. Although these methods have received support from therapists and family associations, their efficiency has not yet been clearly demonstrated empirically and remains a matter of debate [64]. In JeStiMulE, participants had the opportunity to learn progressively a great number of associations, in line with the idea of Kourkoulou *et al*. [65] that ‘restricting learning to a smaller number of stimuli may impede the flexibility with which individuals with ASD can learn new associations’. The results found in the present study suggest that children and adolescents with ASC can learn new associations rapidly and without needing repetition, when they are given the opportunity to use their cognitive strengths to compensate their difficulties in specific areas such as emotion processing. Interestingly, the skills trained with JeStiMulE appear to extend to other stimuli than those included in the training (pictures of real-life characters), suggesting flexible learning and a certain potential of generalization of acquisitions.

### Study limitations and perspectives

ASC include complex and heterogeneous clinical profiles. When studying these disorders, researchers have often to choose to either include a sub-group of individuals with ASC (for example, HFA or LFA) or to include a group of individuals with profiles representative of the entire spectrum. In this study, we have made this latter choice. It allowed us to verify whether JeStiMulE would be a suitable tool for individuals on the whole ASC spectrum rather than for only a sub-group of this spectrum. In order to verify the beneficial effect of JeStiMulE, emotion recognition tasks covering the large spectrum of participants’ intellectual, verbal and academic profiles were proposed to participants. As a consequence, a trade-off between choosing wide-covering tasks and including a significant number of trials per task was necessary. While our results show that heterogeneity does not represent an obstacle to using and benefiting from JeStiMulE, the above mentioned limitations increase results’ variability. In this sense, the study results must be interpreted with precaution. Finally, because the current study was exploratory and aimed first at identifying potential JeStiMulE users, a control group was not included. Further studies including a control group, as well as a larger number of participants representative of the different ASC sub-groups and of different emotion developmental would be of interest to the field. These studies could also focus on the potential of learning transfer from experimental to real-life settings. The relevance of adding physical meaning to emotions (for example, using tactile stimulations) to ASC training programs should also be further explored.

In line with current advances (ASC-Inclusion project [66]), future versions of JeStiMulE are expected to extend to other relevant skills such as imitation and emotion expression (JEMImE [67]). Preliminary findings of our own group have shown that logical processing such as involved in JeStiMulE can also be used to develop academic skills, such word decoding, in children with ASC and without functional language (SEMA-TIC; manuscript in preparation).

## Conclusions

Finally, it is important to note that the aim of this study was not to demonstrate that JeStiMulE can remediate the social impairment, which is a major difficulty at the core of ASC difficulties. ASC have indeed a neurogenetic basis altering brain development and cognitive functioning. This early atypical ‘brain machinery’ alters social perception and social interaction in a very specific way and throughout an individual’s development [68]. Rather than ‘normalized’, social difficulties in ASC may be ‘compensated’ in order to help individuals with LFA and HFA increase their comprehension of the social world and develop strategies to cope with its demands.

## Endnote

^a^Matched validated stimuli could not be found for pain. So, three trials (pictures of Caucasian adults’ faces expressing pain) were created for this emotion.

## Abbreviations

ADI-R: Autism Diagnostic Interview-Revised; ADOS: Autism Diagnostic Observation Schedule; ANOVA: analysis of variance; ASC: Autism Spectrum Conditions; DSM-IV-R: *Diagnostic and Statistical Manual of Mental Diseases, fourth edition*; ECOSSE: Epreuve de COmpréhension Syntaxico-SEmantique; HFA: high-functioning autism; ICT: Information and Communication Technologies; IQ: intelligence quotient; LFA: Low-functioning autism; PDD-NOS: Pervasive Developmental Disorder-Not Otherwise Specified; WASI: Wechsler Abbreviated Scale of Intelligence.

## Competing interests

The authors declare that they have no competing interests.

## Authors’ contributions

SS (first author) was involved in designing the Serious game, conducting the experiment, interpreting data, and drafting the article. SH (second author) was involved in designing the Serious game, conducting the experiment, collecting data and drafting the article. GI was involved in designing the Serious game and conducting the experiment. JL and MA were involved in designing the Serious game, conceptualizing and creating the tactile stimulations and supervising the project. AS was involved in data analyses and interpretation, and drafting the article. SV and FA were involved in interpreting the data. All authors read and approved the final manuscript.

## Supplementary Material

Additional file 1 Detailed participants’ characteristics (in order of increasing age). Click here for file

Additional file 2 **JeStiMulE trailer (**http://www.youtube.com/watch?v=3W-QaLE7hEo&feature=player_embedded**).** Click here for file

Additional file 3 **Data from a preliminary study conducted on typically developing individuals (N = 17).** Results (percentage of correct responses) are presented for each task (table) and then emotion (graphic illustration) separately. Click here for file

Additional file 4 **Number of games completed after the learning phase (N = 11; blue column) and after the training phase (N = 3; red column).** Data is presented for each participant and ranged by age (top) and IQ (bottom). Click here for file

Additional file 5 **Graphic illustration of results before and after four weeks JeStiMulE training.** Data is ranged by participants’ age (top) and IQ (bottom) for each participant in each task. Click here for file
