# Supplementary material for: Facing the challenge of teaching emotions to individuals with low- and high-functioning autism using a new Serious game: a pilot study
Source: Mol Autism. 2014 Jul 1;5:37. doi: 10.1186/2040-2392-5-37 (PMC4094670; doi:10.1186/2040-2392-5-37)
Supplement: Additional file 1 — Detailed participants’ characteristics (in order of increasing age). [file 2040-2392-5-37-S1.pptx]

## Slide 1
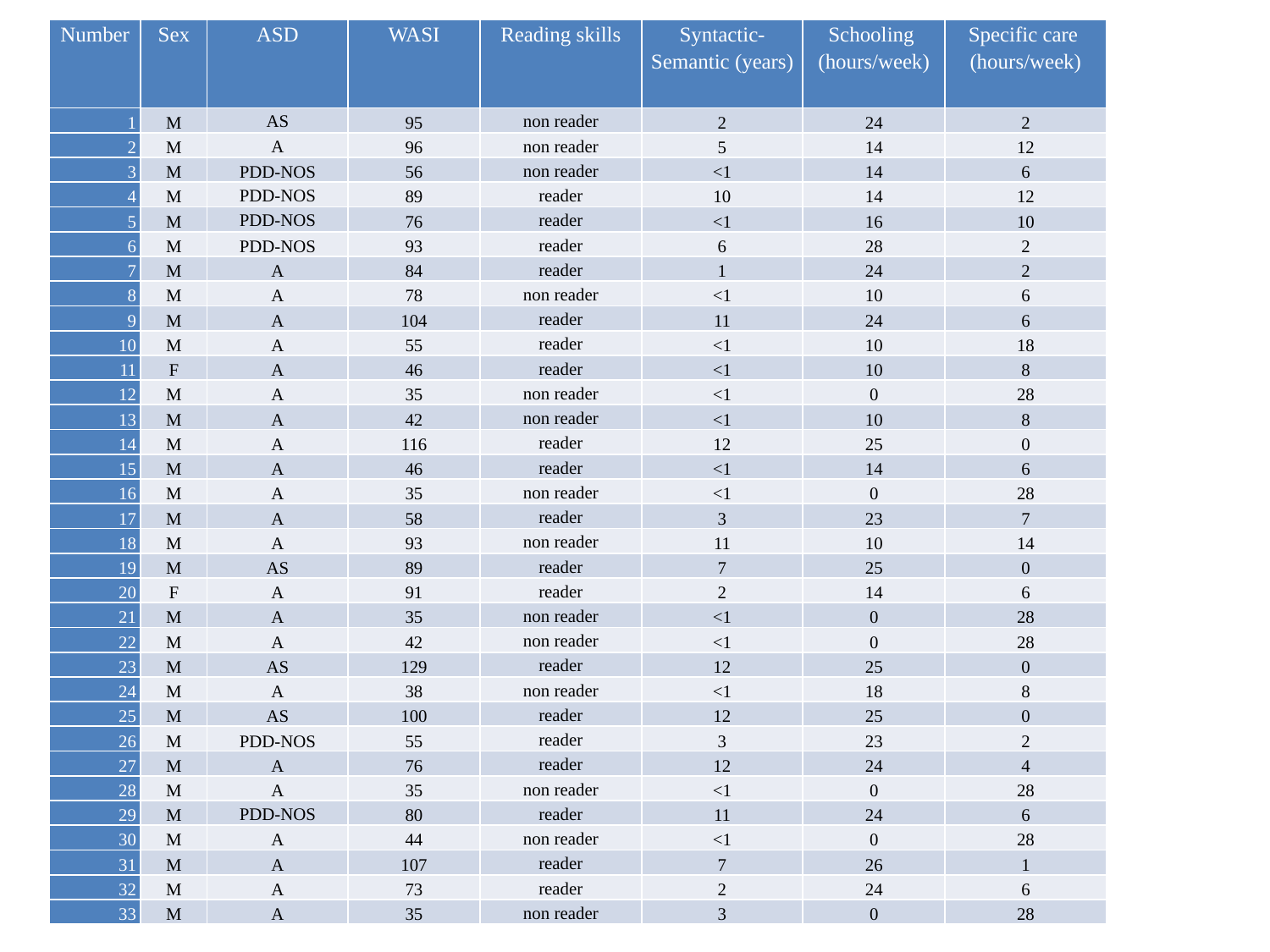

| Number | Sex | ASD | WASI | Reading skills | Syntactic-Semantic (years) | Schooling (hours/week) | Specific care (hours/week) |
| --- | --- | --- | --- | --- | --- | --- | --- |
| 1 | M | AS | 95 | non reader | 2 | 24 | 2 |
| 2 | M | A | 96 | non reader | 5 | 14 | 12 |
| 3 | M | PDD-NOS | 56 | non reader | <1 | 14 | 6 |
| 4 | M | PDD-NOS | 89 | reader | 10 | 14 | 12 |
| 5 | M | PDD-NOS | 76 | reader | <1 | 16 | 10 |
| 6 | M | PDD-NOS | 93 | reader | 6 | 28 | 2 |
| 7 | M | A | 84 | reader | 1 | 24 | 2 |
| 8 | M | A | 78 | non reader | <1 | 10 | 6 |
| 9 | M | A | 104 | reader | 11 | 24 | 6 |
| 10 | M | A | 55 | reader | <1 | 10 | 18 |
| 11 | F | A | 46 | reader | <1 | 10 | 8 |
| 12 | M | A | 35 | non reader | <1 | 0 | 28 |
| 13 | M | A | 42 | non reader | <1 | 10 | 8 |
| 14 | M | A | 116 | reader | 12 | 25 | 0 |
| 15 | M | A | 46 | reader | <1 | 14 | 6 |
| 16 | M | A | 35 | non reader | <1 | 0 | 28 |
| 17 | M | A | 58 | reader | 3 | 23 | 7 |
| 18 | M | A | 93 | non reader | 11 | 10 | 14 |
| 19 | M | AS | 89 | reader | 7 | 25 | 0 |
| 20 | F | A | 91 | reader | 2 | 14 | 6 |
| 21 | M | A | 35 | non reader | <1 | 0 | 28 |
| 22 | M | A | 42 | non reader | <1 | 0 | 28 |
| 23 | M | AS | 129 | reader | 12 | 25 | 0 |
| 24 | M | A | 38 | non reader | <1 | 18 | 8 |
| 25 | M | AS | 100 | reader | 12 | 25 | 0 |
| 26 | M | PDD-NOS | 55 | reader | 3 | 23 | 2 |
| 27 | M | A | 76 | reader | 12 | 24 | 4 |
| 28 | M | A | 35 | non reader | <1 | 0 | 28 |
| 29 | M | PDD-NOS | 80 | reader | 11 | 24 | 6 |
| 30 | M | A | 44 | non reader | <1 | 0 | 28 |
| 31 | M | A | 107 | reader | 7 | 26 | 1 |
| 32 | M | A | 73 | reader | 2 | 24 | 6 |
| 33 | M | A | 35 | non reader | 3 | 0 | 28 |
