# Supplementary material for: Facing the challenge of teaching emotions to individuals with low- and high-functioning autism using a new Serious game: a pilot study
Source: Mol Autism. 2014 Jul 1;5:37. doi: 10.1186/2040-2392-5-37 (PMC4094670; doi:10.1186/2040-2392-5-37)
Supplement: Additional file 2 — JeStiMulE trailer (http://www.youtube.com/watch?v=3W-QaLE7hEo&feature=player_embedded). [file 2040-2392-5-37-S2.doc]

JeStiMulE trailer

<http://www.youtube.com/watch?v=3W-QaLE7hEo&feature=player_embedded>
