# Supplementary material for: Facing the challenge of teaching emotions to individuals with low- and high-functioning autism using a new Serious game: a pilot study
Source: Mol Autism. 2014 Jul 1;5:37. doi: 10.1186/2040-2392-5-37 (PMC4094670; doi:10.1186/2040-2392-5-37)
Supplement: Additional file 3 — Data from a preliminary study conducted on typically developing individuals (N = 17). Results (percentage of correct responses) are presented for each task (table) and then emotion (graphic illustration) separately. [file 2040-2392-5-37-S3.pptx]

## Slide 1
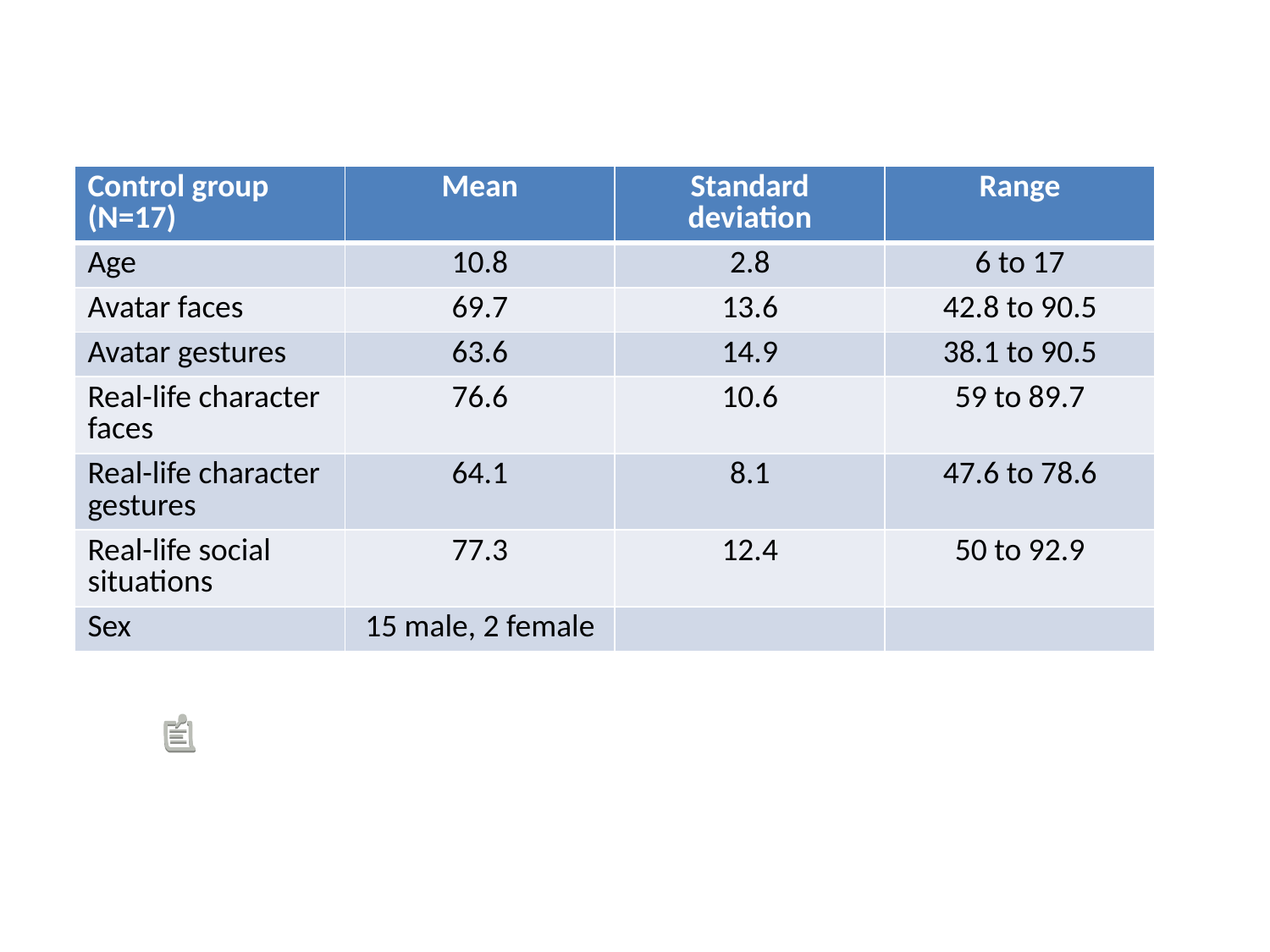

| Control group (N=17) | Mean | Standard deviation | Range |
| --- | --- | --- | --- |
| Age | 10.8 | 2.8 | 6 to 17 |
| Avatar faces | 69.7 | 13.6 | 42.8 to 90.5 |
| Avatar gestures | 63.6 | 14.9 | 38.1 to 90.5 |
| Real-life character faces | 76.6 | 10.6 | 59 to 89.7 |
| Real-life character gestures | 64.1 | 8.1 | 47.6 to 78.6 |
| Real-life social situations | 77.3 | 12.4 | 50 to 92.9 |
| Sex | 15 male, 2 female | | |

## Slide 2
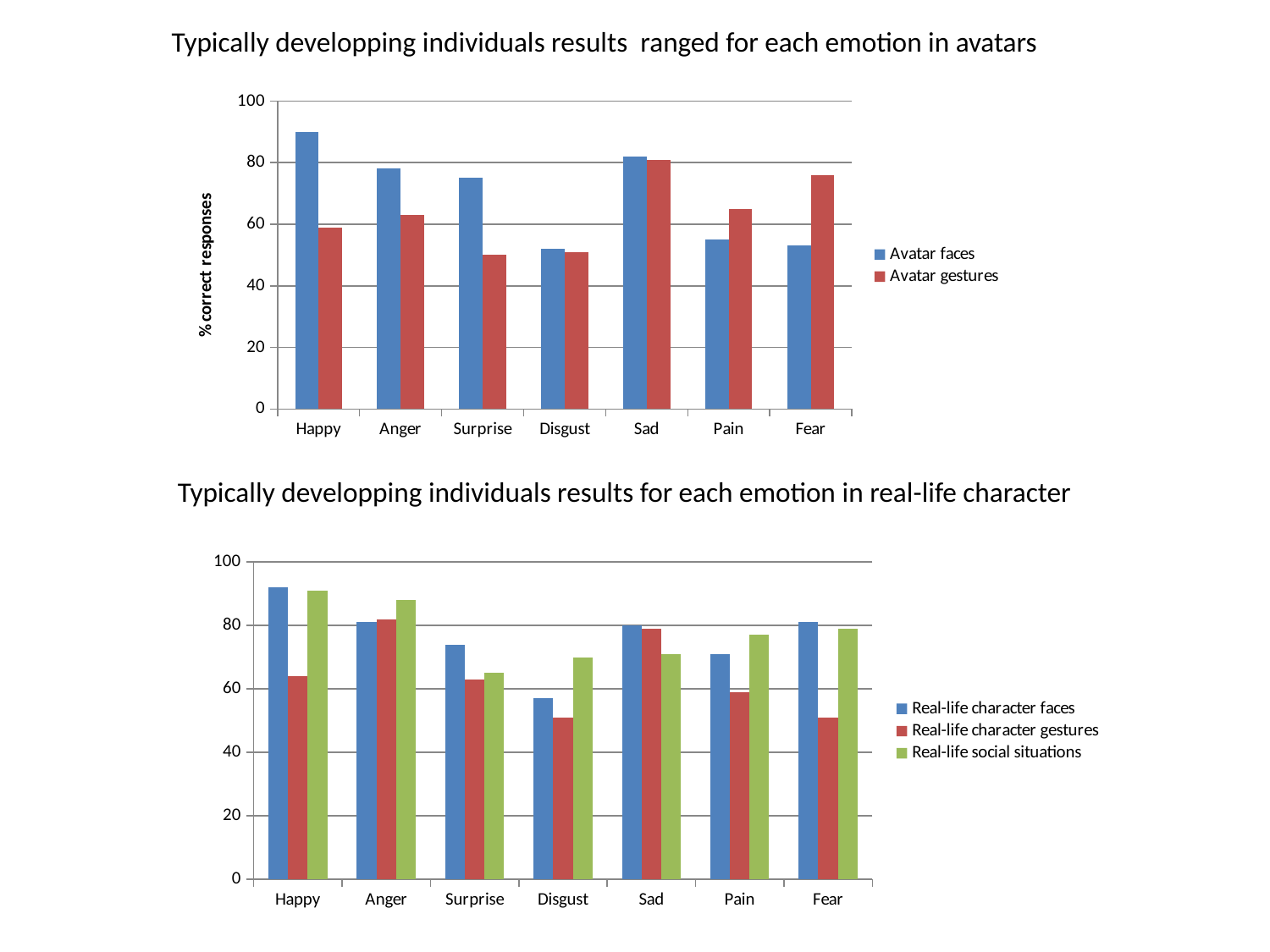

Typically developping individuals results ranged for each emotion in avatars
### Chart
| Category | Avatar faces | Avatar gestures |
|---|---|---|
| Happy | 90.0 | 59.0 |
| Anger | 78.0 | 63.0 |
| Surprise | 75.0 | 50.0 |
| Disgust | 52.0 | 51.0 |
| Sad | 82.0 | 81.0 |
| Pain | 55.0 | 65.0 |
| Fear | 53.0 | 76.0 |Typically developping individuals results for each emotion in real-life character
### Chart
| Category | Real-life character faces | Real-life character gestures | Real-life social situations |
|---|---|---|---|
| Happy | 92.0 | 64.0 | 91.0 |
| Anger | 81.0 | 82.0 | 88.0 |
| Surprise | 74.0 | 63.0 | 65.0 |
| Disgust | 57.0 | 51.0 | 70.0 |
| Sad | 80.0 | 79.0 | 71.0 |
| Pain | 71.0 | 59.0 | 77.0 |
| Fear | 81.0 | 51.0 | 79.0 |
