# Supplementary material for: Facing the challenge of teaching emotions to individuals with low- and high-functioning autism using a new Serious game: a pilot study
Source: Mol Autism. 2014 Jul 1;5:37. doi: 10.1186/2040-2392-5-37 (PMC4094670; doi:10.1186/2040-2392-5-37)
Supplement: Additional file 4 — Number of games completed after the learning phase (N = 11; blue column) and after the training phase (N = 3; red column). Data is presented for each participant and ranged by age (top) and IQ (bottom). [file 2040-2392-5-37-S4.pptx]

## Slide 1
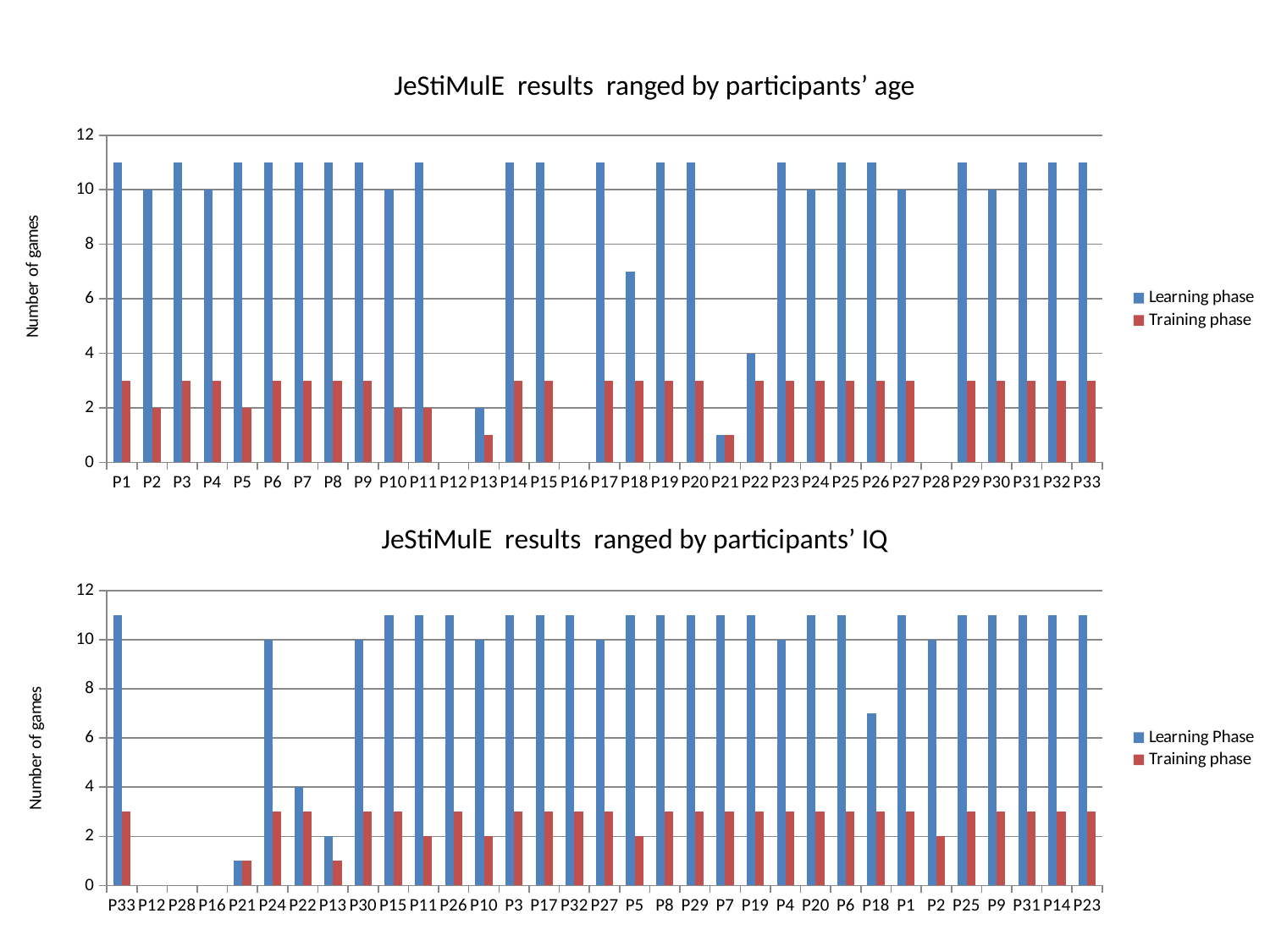

JeStiMulE results ranged by participants’ age
### Chart
| Category | Learning phase | Training phase |
|---|---|---|
| P1 | 11.0 | 3.0 |
| P2 | 10.0 | 2.0 |
| P3 | 11.0 | 3.0 |
| P4 | 10.0 | 3.0 |
| P5 | 11.0 | 2.0 |
| P6 | 11.0 | 3.0 |
| P7 | 11.0 | 3.0 |
| P8 | 11.0 | 3.0 |
| P9 | 11.0 | 3.0 |
| P10 | 10.0 | 2.0 |
| P11 | 11.0 | 2.0 |
| P12 | 0.0 | 0.0 |
| P13 | 2.0 | 1.0 |
| P14 | 11.0 | 3.0 |
| P15 | 11.0 | 3.0 |
| P16 | 0.0 | 0.0 |
| P17 | 11.0 | 3.0 |
| P18 | 7.0 | 3.0 |
| P19 | 11.0 | 3.0 |
| P20 | 11.0 | 3.0 |
| P21 | 1.0 | 1.0 |
| P22 | 4.0 | 3.0 |
| P23 | 11.0 | 3.0 |
| P24 | 10.0 | 3.0 |
| P25 | 11.0 | 3.0 |
| P26 | 11.0 | 3.0 |
| P27 | 10.0 | 3.0 |
| P28 | 0.0 | 0.0 |
| P29 | 11.0 | 3.0 |
| P30 | 10.0 | 3.0 |
| P31 | 11.0 | 3.0 |
| P32 | 11.0 | 3.0 |
| P33 | 11.0 | 3.0 |JeStiMulE results ranged by participants’ IQ
### Chart
| Category | Learning Phase | Training phase |
|---|---|---|
| P33 | 11.0 | 3.0 |
| P12 | 0.0 | 0.0 |
| P28 | 0.0 | 0.0 |
| P16 | 0.0 | 0.0 |
| P21 | 1.0 | 1.0 |
| P24 | 10.0 | 3.0 |
| P22 | 4.0 | 3.0 |
| P13 | 2.0 | 1.0 |
| P30 | 10.0 | 3.0 |
| P15 | 11.0 | 3.0 |
| P11 | 11.0 | 2.0 |
| P26 | 11.0 | 3.0 |
| P10 | 10.0 | 2.0 |
| P3 | 11.0 | 3.0 |
| P17 | 11.0 | 3.0 |
| P32 | 11.0 | 3.0 |
| P27 | 10.0 | 3.0 |
| P5 | 11.0 | 2.0 |
| P8 | 11.0 | 3.0 |
| P29 | 11.0 | 3.0 |
| P7 | 11.0 | 3.0 |
| P19 | 11.0 | 3.0 |
| P4 | 10.0 | 3.0 |
| P20 | 11.0 | 3.0 |
| P6 | 11.0 | 3.0 |
| P18 | 7.0 | 3.0 |
| P1 | 11.0 | 3.0 |
| P2 | 10.0 | 2.0 |
| P25 | 11.0 | 3.0 |
| P9 | 11.0 | 3.0 |
| P31 | 11.0 | 3.0 |
| P14 | 11.0 | 3.0 |
| P23 | 11.0 | 3.0 |
