# Supplementary material for: Facing the challenge of teaching emotions to individuals with low- and high-functioning autism using a new Serious game: a pilot study
Source: Mol Autism. 2014 Jul 1;5:37. doi: 10.1186/2040-2392-5-37 (PMC4094670; doi:10.1186/2040-2392-5-37)
Supplement: Additional file 5 — Graphic illustration of results before and after four weeks JeStiMulE training. Data is ranged by participants’ age (top) and IQ (bottom) for each participant in each task. [file 2040-2392-5-37-S5.pptx]

## Slide 1
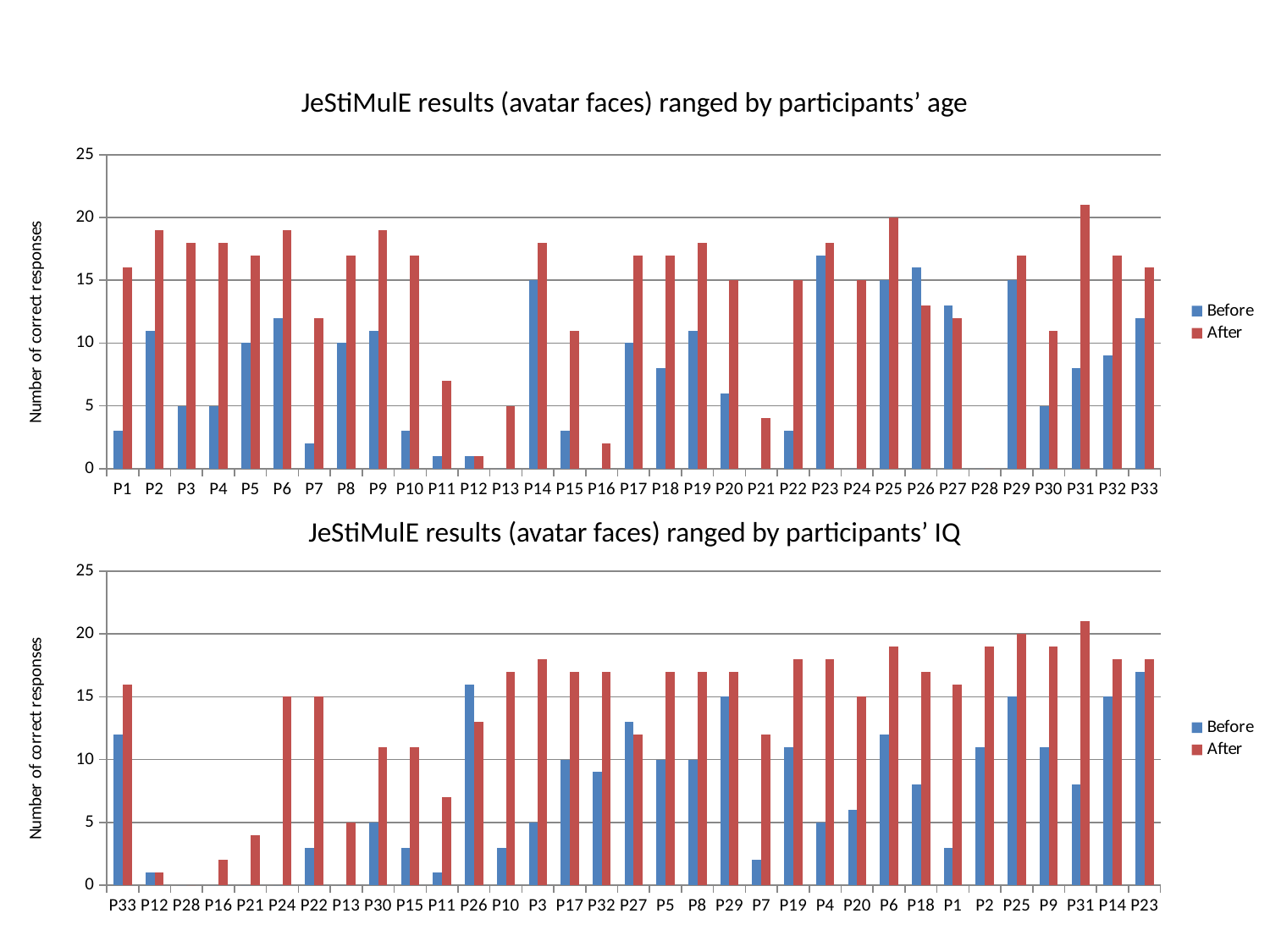

JeStiMulE results (avatar faces) ranged by participants’ age
### Chart
| Category | Before | After |
|---|---|---|
| P1 | 3.0 | 16.0 |
| P2 | 11.0 | 19.0 |
| P3 | 5.0 | 18.0 |
| P4 | 5.0 | 18.0 |
| P5 | 10.0 | 17.0 |
| P6 | 12.0 | 19.0 |
| P7 | 2.0 | 12.0 |
| P8 | 10.0 | 17.0 |
| P9 | 11.0 | 19.0 |
| P10 | 3.0 | 17.0 |
| P11 | 1.0 | 7.0 |
| P12 | 1.0 | 1.0 |
| P13 | 0.0 | 5.0 |
| P14 | 15.0 | 18.0 |
| P15 | 3.0 | 11.0 |
| P16 | 0.0 | 2.0 |
| P17 | 10.0 | 17.0 |
| P18 | 8.0 | 17.0 |
| P19 | 11.0 | 18.0 |
| P20 | 6.0 | 15.0 |
| P21 | 0.0 | 4.0 |
| P22 | 3.0 | 15.0 |
| P23 | 17.0 | 18.0 |
| P24 | 0.0 | 15.0 |
| P25 | 15.0 | 20.0 |
| P26 | 16.0 | 13.0 |
| P27 | 13.0 | 12.0 |
| P28 | 0.0 | 0.0 |
| P29 | 15.0 | 17.0 |
| P30 | 5.0 | 11.0 |
| P31 | 8.0 | 21.0 |
| P32 | 9.0 | 17.0 |
| P33 | 12.0 | 16.0 |JeStiMulE results (avatar faces) ranged by participants’ IQ
### Chart
| Category | Before | After |
|---|---|---|
| P33 | 12.0 | 16.0 |
| P12 | 1.0 | 1.0 |
| P28 | 0.0 | 0.0 |
| P16 | 0.0 | 2.0 |
| P21 | 0.0 | 4.0 |
| P24 | 0.0 | 15.0 |
| P22 | 3.0 | 15.0 |
| P13 | 0.0 | 5.0 |
| P30 | 5.0 | 11.0 |
| P15 | 3.0 | 11.0 |
| P11 | 1.0 | 7.0 |
| P26 | 16.0 | 13.0 |
| P10 | 3.0 | 17.0 |
| P3 | 5.0 | 18.0 |
| P17 | 10.0 | 17.0 |
| P32 | 9.0 | 17.0 |
| P27 | 13.0 | 12.0 |
| P5 | 10.0 | 17.0 |
| P8 | 10.0 | 17.0 |
| P29 | 15.0 | 17.0 |
| P7 | 2.0 | 12.0 |
| P19 | 11.0 | 18.0 |
| P4 | 5.0 | 18.0 |
| P20 | 6.0 | 15.0 |
| P6 | 12.0 | 19.0 |
| P18 | 8.0 | 17.0 |
| P1 | 3.0 | 16.0 |
| P2 | 11.0 | 19.0 |
| P25 | 15.0 | 20.0 |
| P9 | 11.0 | 19.0 |
| P31 | 8.0 | 21.0 |
| P14 | 15.0 | 18.0 |
| P23 | 17.0 | 18.0 |

## Slide 2
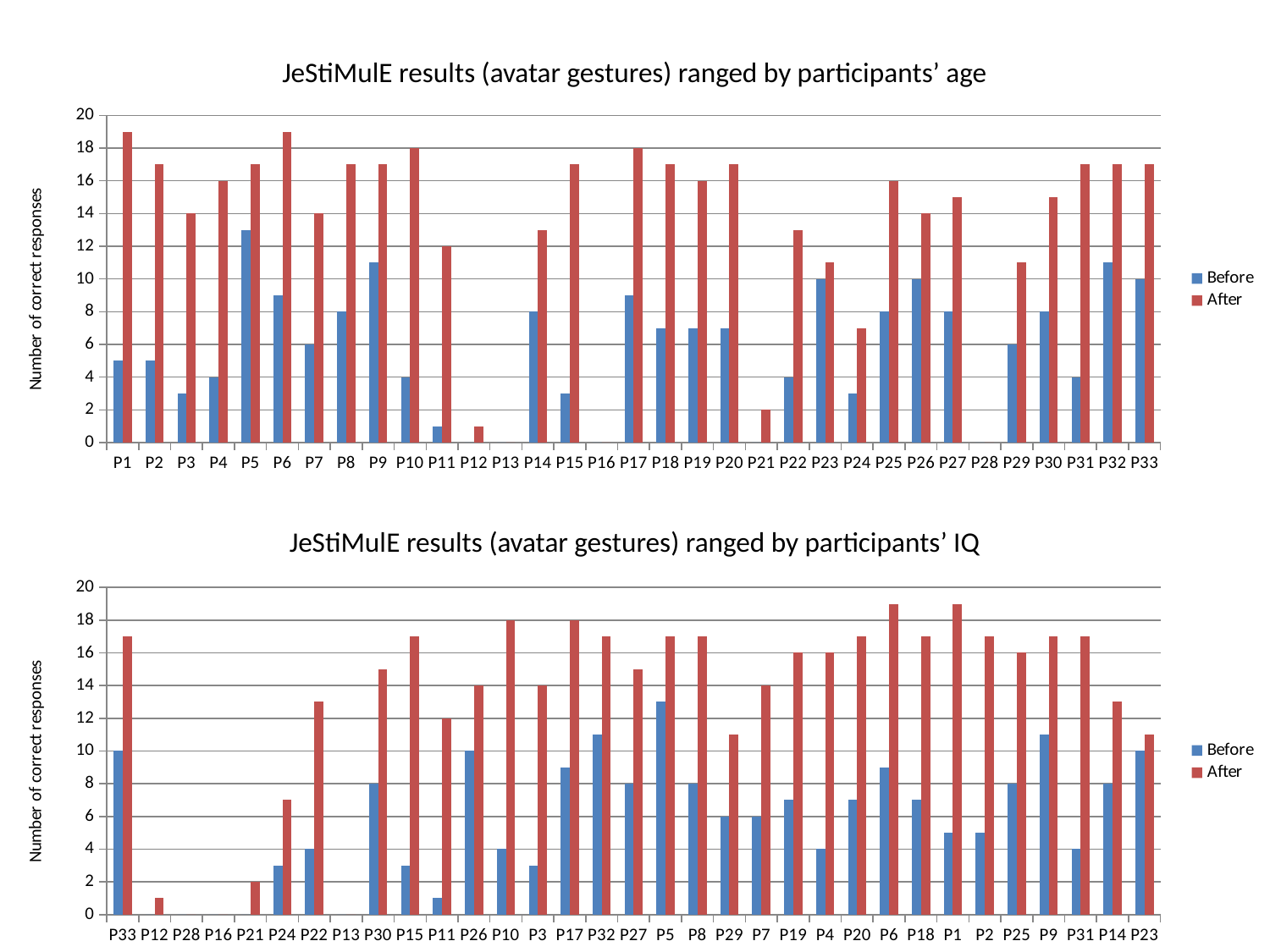

JeStiMulE results (avatar gestures) ranged by participants’ age
### Chart
| Category | Before | After |
|---|---|---|
| P1 | 5.0 | 19.0 |
| P2 | 5.0 | 17.0 |
| P3 | 3.0 | 14.0 |
| P4 | 4.0 | 16.0 |
| P5 | 13.0 | 17.0 |
| P6 | 9.0 | 19.0 |
| P7 | 6.0 | 14.0 |
| P8 | 8.0 | 17.0 |
| P9 | 11.0 | 17.0 |
| P10 | 4.0 | 18.0 |
| P11 | 1.0 | 12.0 |
| P12 | 0.0 | 1.0 |
| P13 | 0.0 | 0.0 |
| P14 | 8.0 | 13.0 |
| P15 | 3.0 | 17.0 |
| P16 | 0.0 | 0.0 |
| P17 | 9.0 | 18.0 |
| P18 | 7.0 | 17.0 |
| P19 | 7.0 | 16.0 |
| P20 | 7.0 | 17.0 |
| P21 | 0.0 | 2.0 |
| P22 | 4.0 | 13.0 |
| P23 | 10.0 | 11.0 |
| P24 | 3.0 | 7.0 |
| P25 | 8.0 | 16.0 |
| P26 | 10.0 | 14.0 |
| P27 | 8.0 | 15.0 |
| P28 | 0.0 | 0.0 |
| P29 | 6.0 | 11.0 |
| P30 | 8.0 | 15.0 |
| P31 | 4.0 | 17.0 |
| P32 | 11.0 | 17.0 |
| P33 | 10.0 | 17.0 |JeStiMulE results (avatar gestures) ranged by participants’ IQ
### Chart
| Category | Before | After |
|---|---|---|
| P33 | 10.0 | 17.0 |
| P12 | 0.0 | 1.0 |
| P28 | 0.0 | 0.0 |
| P16 | 0.0 | 0.0 |
| P21 | 0.0 | 2.0 |
| P24 | 3.0 | 7.0 |
| P22 | 4.0 | 13.0 |
| P13 | 0.0 | 0.0 |
| P30 | 8.0 | 15.0 |
| P15 | 3.0 | 17.0 |
| P11 | 1.0 | 12.0 |
| P26 | 10.0 | 14.0 |
| P10 | 4.0 | 18.0 |
| P3 | 3.0 | 14.0 |
| P17 | 9.0 | 18.0 |
| P32 | 11.0 | 17.0 |
| P27 | 8.0 | 15.0 |
| P5 | 13.0 | 17.0 |
| P8 | 8.0 | 17.0 |
| P29 | 6.0 | 11.0 |
| P7 | 6.0 | 14.0 |
| P19 | 7.0 | 16.0 |
| P4 | 4.0 | 16.0 |
| P20 | 7.0 | 17.0 |
| P6 | 9.0 | 19.0 |
| P18 | 7.0 | 17.0 |
| P1 | 5.0 | 19.0 |
| P2 | 5.0 | 17.0 |
| P25 | 8.0 | 16.0 |
| P9 | 11.0 | 17.0 |
| P31 | 4.0 | 17.0 |
| P14 | 8.0 | 13.0 |
| P23 | 10.0 | 11.0 |

## Slide 3
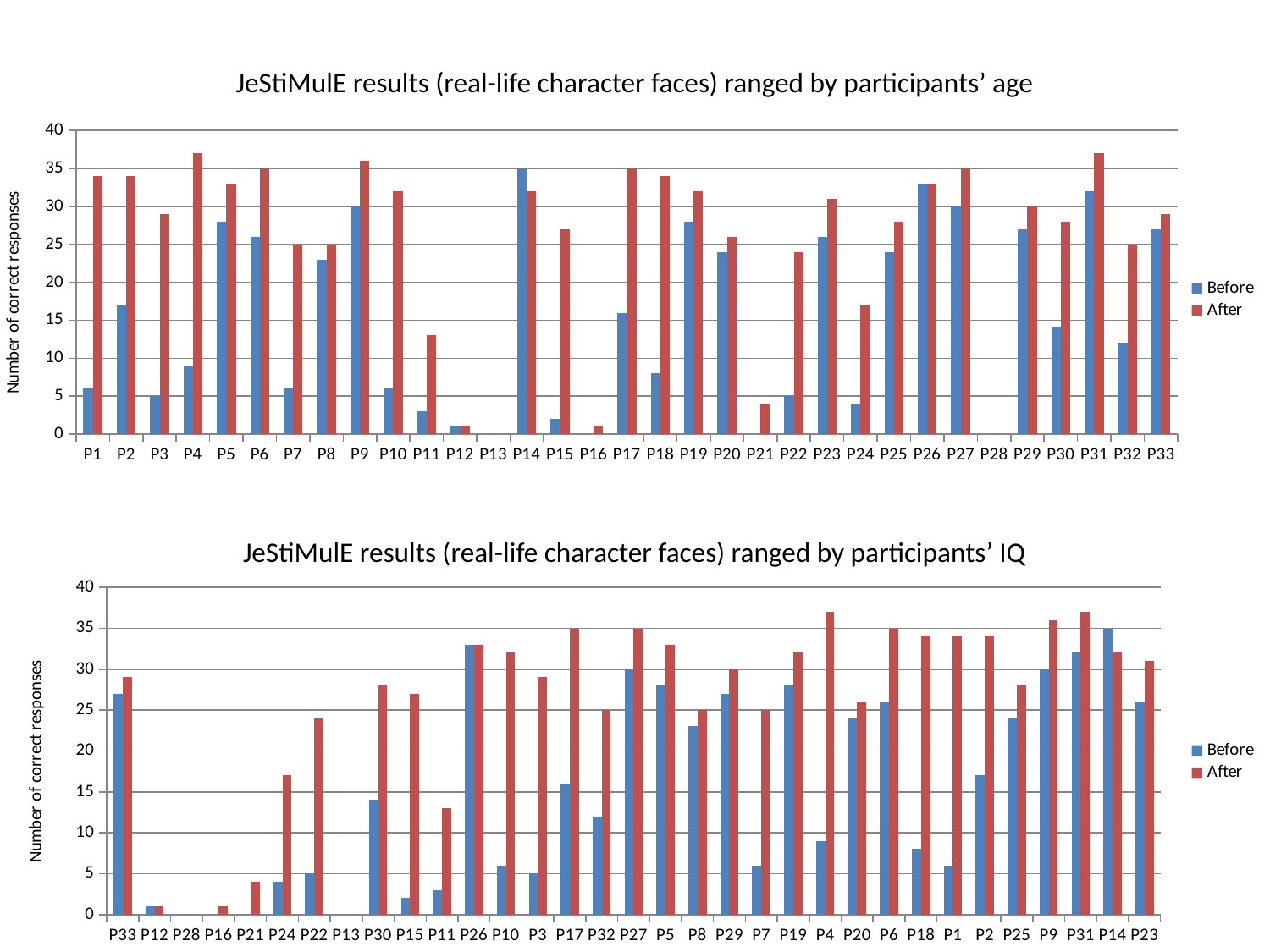

JeStiMulE results (real-life character faces) ranged by participants’ age
### Chart
| Category | Before | After |
|---|---|---|
| P1 | 6.0 | 34.0 |
| P2 | 17.0 | 34.0 |
| P3 | 5.0 | 29.0 |
| P4 | 9.0 | 37.0 |
| P5 | 28.0 | 33.0 |
| P6 | 26.0 | 35.0 |
| P7 | 6.0 | 25.0 |
| P8 | 23.0 | 25.0 |
| P9 | 30.0 | 36.0 |
| P10 | 6.0 | 32.0 |
| P11 | 3.0 | 13.0 |
| P12 | 1.0 | 1.0 |
| P13 | 0.0 | 0.0 |
| P14 | 35.0 | 32.0 |
| P15 | 2.0 | 27.0 |
| P16 | 0.0 | 1.0 |
| P17 | 16.0 | 35.0 |
| P18 | 8.0 | 34.0 |
| P19 | 28.0 | 32.0 |
| P20 | 24.0 | 26.0 |
| P21 | 0.0 | 4.0 |
| P22 | 5.0 | 24.0 |
| P23 | 26.0 | 31.0 |
| P24 | 4.0 | 17.0 |
| P25 | 24.0 | 28.0 |
| P26 | 33.0 | 33.0 |
| P27 | 30.0 | 35.0 |
| P28 | 0.0 | 0.0 |
| P29 | 27.0 | 30.0 |
| P30 | 14.0 | 28.0 |
| P31 | 32.0 | 37.0 |
| P32 | 12.0 | 25.0 |
| P33 | 27.0 | 29.0 |JeStiMulE results (real-life character faces) ranged by participants’ IQ
### Chart
| Category | Before | After |
|---|---|---|
| P33 | 27.0 | 29.0 |
| P12 | 1.0 | 1.0 |
| P28 | 0.0 | 0.0 |
| P16 | 0.0 | 1.0 |
| P21 | 0.0 | 4.0 |
| P24 | 4.0 | 17.0 |
| P22 | 5.0 | 24.0 |
| P13 | 0.0 | 0.0 |
| P30 | 14.0 | 28.0 |
| P15 | 2.0 | 27.0 |
| P11 | 3.0 | 13.0 |
| P26 | 33.0 | 33.0 |
| P10 | 6.0 | 32.0 |
| P3 | 5.0 | 29.0 |
| P17 | 16.0 | 35.0 |
| P32 | 12.0 | 25.0 |
| P27 | 30.0 | 35.0 |
| P5 | 28.0 | 33.0 |
| P8 | 23.0 | 25.0 |
| P29 | 27.0 | 30.0 |
| P7 | 6.0 | 25.0 |
| P19 | 28.0 | 32.0 |
| P4 | 9.0 | 37.0 |
| P20 | 24.0 | 26.0 |
| P6 | 26.0 | 35.0 |
| P18 | 8.0 | 34.0 |
| P1 | 6.0 | 34.0 |
| P2 | 17.0 | 34.0 |
| P25 | 24.0 | 28.0 |
| P9 | 30.0 | 36.0 |
| P31 | 32.0 | 37.0 |
| P14 | 35.0 | 32.0 |
| P23 | 26.0 | 31.0 |

## Slide 4
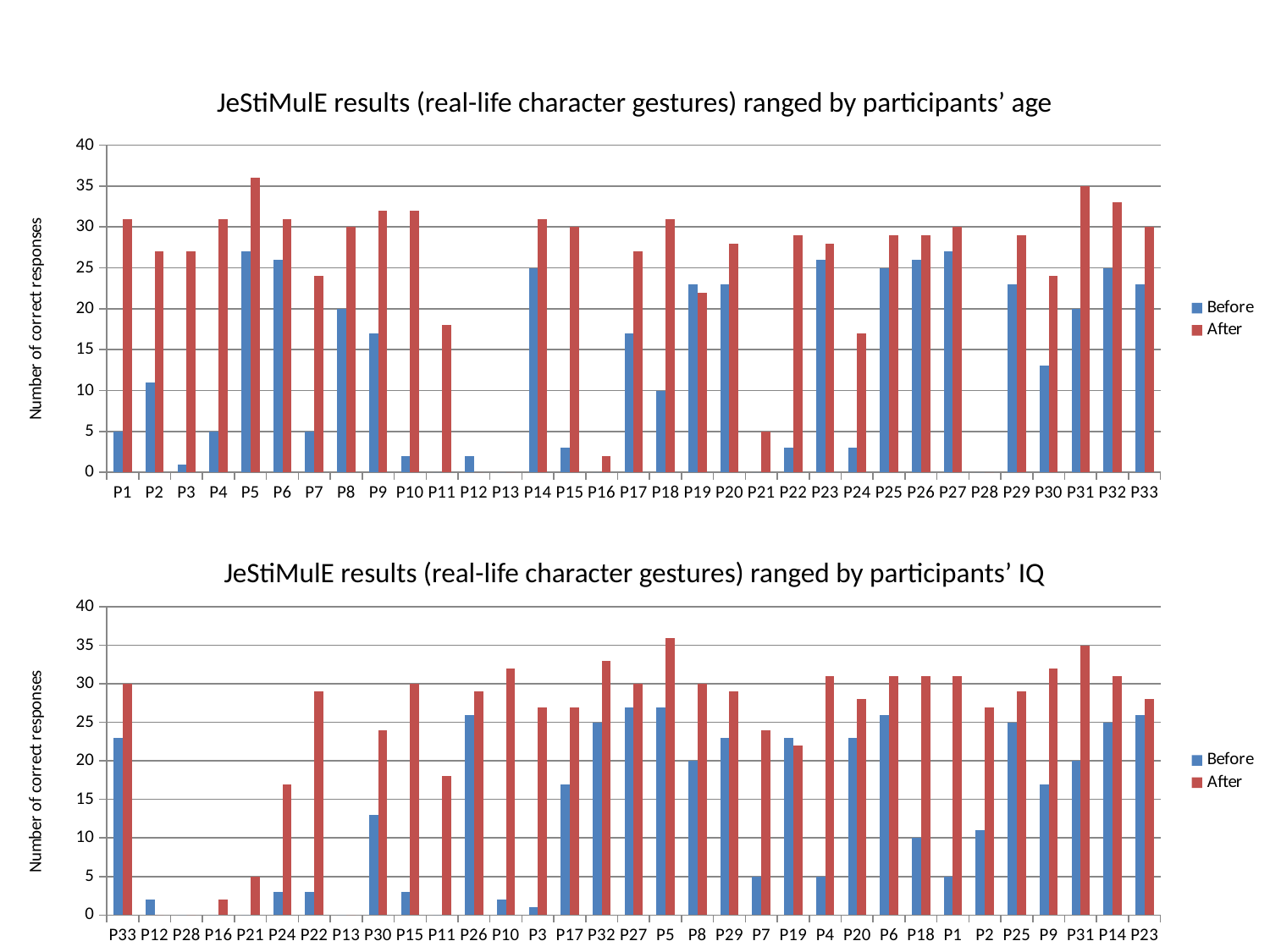

JeStiMulE results (real-life character gestures) ranged by participants’ age
### Chart
| Category | Before | After |
|---|---|---|
| P1 | 5.0 | 31.0 |
| P2 | 11.0 | 27.0 |
| P3 | 1.0 | 27.0 |
| P4 | 5.0 | 31.0 |
| P5 | 27.0 | 36.0 |
| P6 | 26.0 | 31.0 |
| P7 | 5.0 | 24.0 |
| P8 | 20.0 | 30.0 |
| P9 | 17.0 | 32.0 |
| P10 | 2.0 | 32.0 |
| P11 | 0.0 | 18.0 |
| P12 | 2.0 | 0.0 |
| P13 | 0.0 | 0.0 |
| P14 | 25.0 | 31.0 |
| P15 | 3.0 | 30.0 |
| P16 | 0.0 | 2.0 |
| P17 | 17.0 | 27.0 |
| P18 | 10.0 | 31.0 |
| P19 | 23.0 | 22.0 |
| P20 | 23.0 | 28.0 |
| P21 | 0.0 | 5.0 |
| P22 | 3.0 | 29.0 |
| P23 | 26.0 | 28.0 |
| P24 | 3.0 | 17.0 |
| P25 | 25.0 | 29.0 |
| P26 | 26.0 | 29.0 |
| P27 | 27.0 | 30.0 |
| P28 | 0.0 | 0.0 |
| P29 | 23.0 | 29.0 |
| P30 | 13.0 | 24.0 |
| P31 | 20.0 | 35.0 |
| P32 | 25.0 | 33.0 |
| P33 | 23.0 | 30.0 |JeStiMulE results (real-life character gestures) ranged by participants’ IQ
### Chart
| Category | Before | After |
|---|---|---|
| P33 | 23.0 | 30.0 |
| P12 | 2.0 | 0.0 |
| P28 | 0.0 | 0.0 |
| P16 | 0.0 | 2.0 |
| P21 | 0.0 | 5.0 |
| P24 | 3.0 | 17.0 |
| P22 | 3.0 | 29.0 |
| P13 | 0.0 | 0.0 |
| P30 | 13.0 | 24.0 |
| P15 | 3.0 | 30.0 |
| P11 | 0.0 | 18.0 |
| P26 | 26.0 | 29.0 |
| P10 | 2.0 | 32.0 |
| P3 | 1.0 | 27.0 |
| P17 | 17.0 | 27.0 |
| P32 | 25.0 | 33.0 |
| P27 | 27.0 | 30.0 |
| P5 | 27.0 | 36.0 |
| P8 | 20.0 | 30.0 |
| P29 | 23.0 | 29.0 |
| P7 | 5.0 | 24.0 |
| P19 | 23.0 | 22.0 |
| P4 | 5.0 | 31.0 |
| P20 | 23.0 | 28.0 |
| P6 | 26.0 | 31.0 |
| P18 | 10.0 | 31.0 |
| P1 | 5.0 | 31.0 |
| P2 | 11.0 | 27.0 |
| P25 | 25.0 | 29.0 |
| P9 | 17.0 | 32.0 |
| P31 | 20.0 | 35.0 |
| P14 | 25.0 | 31.0 |
| P23 | 26.0 | 28.0 |

## Slide 5
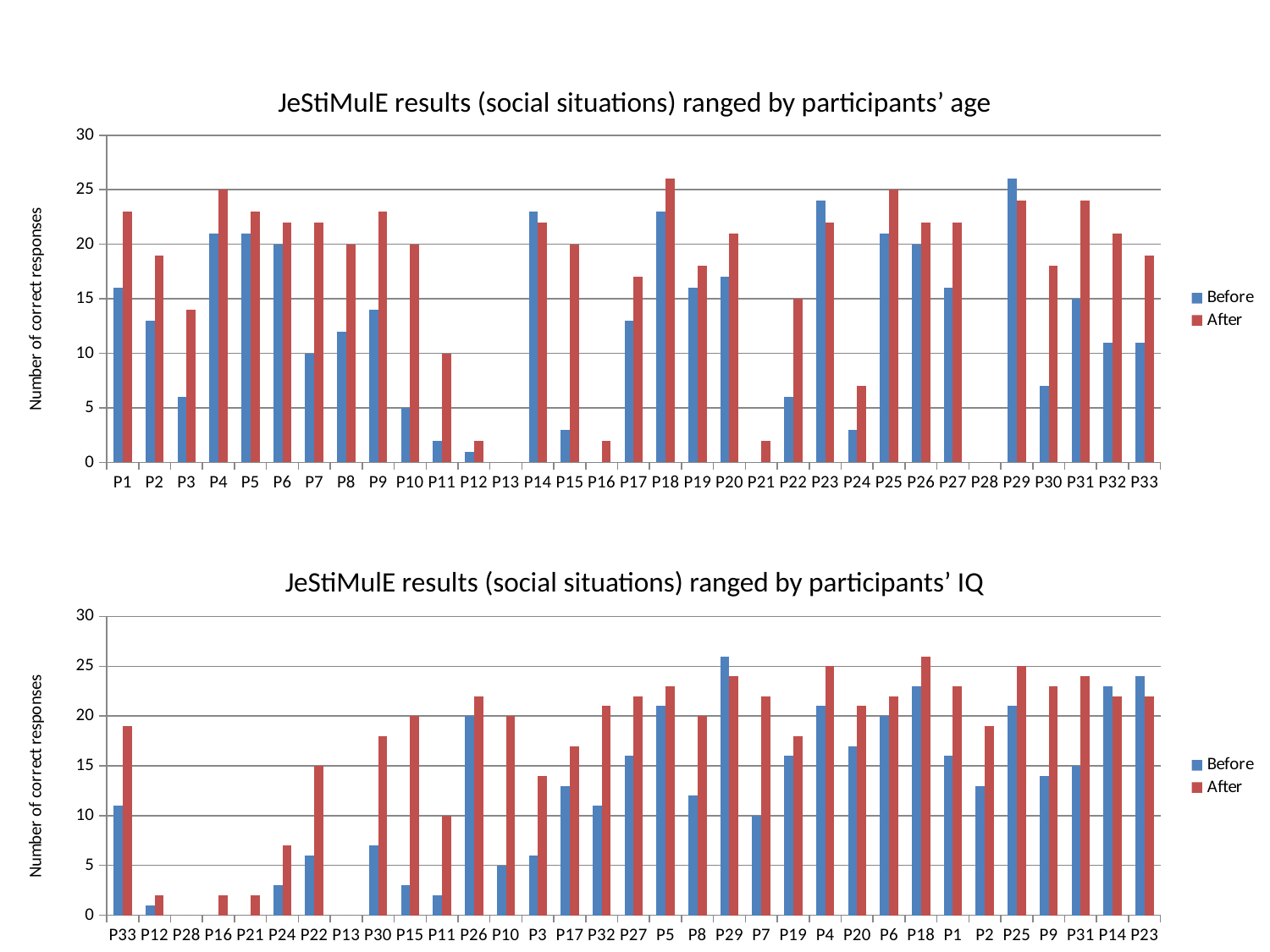

JeStiMulE results (social situations) ranged by participants’ age
### Chart
| Category | Before | After |
|---|---|---|
| P1 | 16.0 | 23.0 |
| P2 | 13.0 | 19.0 |
| P3 | 6.0 | 14.0 |
| P4 | 21.0 | 25.0 |
| P5 | 21.0 | 23.0 |
| P6 | 20.0 | 22.0 |
| P7 | 10.0 | 22.0 |
| P8 | 12.0 | 20.0 |
| P9 | 14.0 | 23.0 |
| P10 | 5.0 | 20.0 |
| P11 | 2.0 | 10.0 |
| P12 | 1.0 | 2.0 |
| P13 | 0.0 | 0.0 |
| P14 | 23.0 | 22.0 |
| P15 | 3.0 | 20.0 |
| P16 | 0.0 | 2.0 |
| P17 | 13.0 | 17.0 |
| P18 | 23.0 | 26.0 |
| P19 | 16.0 | 18.0 |
| P20 | 17.0 | 21.0 |
| P21 | 0.0 | 2.0 |
| P22 | 6.0 | 15.0 |
| P23 | 24.0 | 22.0 |
| P24 | 3.0 | 7.0 |
| P25 | 21.0 | 25.0 |
| P26 | 20.0 | 22.0 |
| P27 | 16.0 | 22.0 |
| P28 | 0.0 | 0.0 |
| P29 | 26.0 | 24.0 |
| P30 | 7.0 | 18.0 |
| P31 | 15.0 | 24.0 |
| P32 | 11.0 | 21.0 |
| P33 | 11.0 | 19.0 |JeStiMulE results (social situations) ranged by participants’ IQ
### Chart
| Category | Before | After |
|---|---|---|
| P33 | 11.0 | 19.0 |
| P12 | 1.0 | 2.0 |
| P28 | 0.0 | 0.0 |
| P16 | 0.0 | 2.0 |
| P21 | 0.0 | 2.0 |
| P24 | 3.0 | 7.0 |
| P22 | 6.0 | 15.0 |
| P13 | 0.0 | 0.0 |
| P30 | 7.0 | 18.0 |
| P15 | 3.0 | 20.0 |
| P11 | 2.0 | 10.0 |
| P26 | 20.0 | 22.0 |
| P10 | 5.0 | 20.0 |
| P3 | 6.0 | 14.0 |
| P17 | 13.0 | 17.0 |
| P32 | 11.0 | 21.0 |
| P27 | 16.0 | 22.0 |
| P5 | 21.0 | 23.0 |
| P8 | 12.0 | 20.0 |
| P29 | 26.0 | 24.0 |
| P7 | 10.0 | 22.0 |
| P19 | 16.0 | 18.0 |
| P4 | 21.0 | 25.0 |
| P20 | 17.0 | 21.0 |
| P6 | 20.0 | 22.0 |
| P18 | 23.0 | 26.0 |
| P1 | 16.0 | 23.0 |
| P2 | 13.0 | 19.0 |
| P25 | 21.0 | 25.0 |
| P9 | 14.0 | 23.0 |
| P31 | 15.0 | 24.0 |
| P14 | 23.0 | 22.0 |
| P23 | 24.0 | 22.0 |
